# The Effects of Glaucoma on the Pressure-Induced Strain Response of the Human Lamina Cribrosa

**DOI:** 10.1167/iovs.61.4.41

**Published:** 2020-04-28

**Authors:** Dan Midgett, Baiyun Liu, Yik Tung Tracy Ling, Joan L. Jefferys, Harry A. Quigley, Thao D. Nguyen

**Affiliations:** 1 Department of Mechanical Engineering, The Johns Hopkins University, Baltimore, Maryland, United States; 2 Wilmer Ophthalmological Institute, School of Medicine, The Johns Hopkins University, Baltimore, Maryland, United States; 3 Department of Materials Science, The Johns Hopkins University, Baltimore, Maryland, United States

**Keywords:** glaucoma, lamina cribrosa, optic nerve head, biomechanics, intraocular pressure

## Abstract

**Purpose:**

To measure the ex vivo pressure-induced strain response of the human optic nerve head and analyze for variations with glaucoma diagnosis and optic nerve axon damage.

**Methods:**

The posterior sclera of 16 eyes from 8 diagnosed glaucoma donors and 10 eyes from 6 donors with no history of glaucoma were inflation tested between 5 and 45 mm Hg. The optic nerve from each donor was examined for degree of axon loss. The posterior volume of the lamina cribrosa (LC) was imaged with second harmonic generation and analyzed using volume correlation to calculate LC strains between 5 and 10 and 5 and 45 mm Hg.

**Results:**

Eye length and LC area were larger in eyes diagnosed with glaucoma (*P* *≤* 0*.*03). Nasal-temporal *E_XX_* and circumferential *E_θθ_* strains were lower in the LC of diagnosed glaucoma eyes at 10 mm Hg (*P* *≤* 0*.*05) and 45 mm Hg (*P* *≤* 0*.*07). *E_XX_* was smaller in the LC of glaucoma eyes with <25% axon loss compared with undamaged normal eyes (*P* = 0*.*01, 45 mm Hg). In general, the strains were larger in the peripheral than central LC. The ratio of the maximum principal strain *E_max_* in the peripheral to central LC was larger in glaucoma eyes with >25% axon loss than in glaucoma eyes with milder damage (*P* = 0*.*004, 10 mm Hg).

**Conclusions:**

The stiffness of the LC pressure-strain response was greater in diagnosed glaucoma eyes and varied with glaucomatous axon damage. Lower LC strains in glaucoma eyes with milder damage may represent baseline biomechanical behavior that contributes to axon loss, whereas greater LC strain and altered radial LC strain variation in glaucoma eyes with more severe damage may be caused by glaucoma-related remodeling.

Glaucoma is a neurodegenerative disease characterized by the dysfunction and death of retinal ganglion cell (RGC) axons at the lamina cribrosa (LC) in the optic nerve head (ONH). This is accompanied by significant remodeling of the connective tissue structure of the LC, which gives the optic disk in advanced glaucoma patients a more excavated appearance.^1^^,^^2^ The level of intraocular pressure (IOP) is an important risk factor that correlates with the prevalence of glaucoma and the severity of glaucomatous axon damage.^3^^,^^4^ IOP acts to deform the tissues of the ONH by imposing a translaminar pressure difference and inducing tensile hoop stresses in the adjacent sclera. The LC is the main load-bearing tissue structure of the ONH that serves to support the RGC axons as they pass from the intraocular space into the optic nerve. The collagen beams of the LC also house mechanosensitive astrocytes, fibroblast-like cells called lamina cribrocytes, and microglia, as well as nourishing capillaries.^5^ The biomechanical response of the LC to IOP fluctuations may regulate the homoeostasis in the ONH.^6^^,^^7^ Changes in the structure and mechanical properties of the LC may alter the mechanical and physiological support of the RCG axons and contribute to the susceptibility and severity of glaucomatous axon damage. Variations in the LC structure and stiffness may explain why some ocular hypertensives do not develop glaucoma while others with normal or low IOP develop glaucoma. Advances in volumetric imaging methods, such as optical coherence tomography (OCT) and multiphoton microscopy, and volume correlation methods have enabled direct, spatially resolved measurements of ONH deformation in response to changes in IOP in human,^8^^–^^13^ mouse,^14^^,^^15^ and porcine eye,^16^ and to changes in IOP and intracranial pressure in monkey eyes.^10^^,^^17^ Midgett et al.^9^ developed an ex vivo inflation test that used second harmonic generation (SHG) imaging of collagen in the posterior LC volume and digital volume correlation (DVC) to measure the strain response of the human LC to controlled pressurization. LC strains were larger in the peripheral LC compared to the central LC. Comparing the nasal, temporal, inferior, and superior LC quadrants, maximum principal strain was lowest in the nasal quadrant. Specimen-averaged maximum principal strain also decreased significantly with age, suggesting a structural stiffening with age. Behkam et al.^13^ developed a different inflation test that also used SHG volume imaging and DVC to measure the pressure-strain response of the human LC and compared for differences between different racioethnic groups. They found significant differences in the shear strains and regional variation of the strain components in the LC between Hispanic, African-derived, and European-derived racial groups. Girard et al.^8^ used OCT to image the visible anterior portion of the ONH in patients before and after trabeculectomy and applied DVC to calculate strain relief after the IOP-lowering surgery. Beotra et al.^12^ applied the same methods to measure LC strains following acute IOP elevation by an ophthalmodynamometer in healthy, ocular hypertensive, and glaucoma subjects. Effective LC strain in subjects with ocular hypertension was significantly smaller than in healthy subjects, but was not significantly different compared with glaucoma subjects. There were also no significant differences in LC strain between patients with primary open angle glaucoma (POAG) and angle closure glaucoma (ACG). These studies have highlighted average differences in the LC pressure-strain response of glaucoma eyes, but so far no study has examined how regional strain distribution within the LC differs between glaucoma and healthy eyes and with the degree of axonal damage.

The objective of this study is to measure the IOP-induced deformation of the LC in postmortem normal and glaucoma donor eyes and analyze for variations with glaucoma diagnosis and optic nerve axon damage. The ex vivo inflation test method developed by Midgett et al.^9^ was applied to measure strains in the LC between the pressures of 5 to 10 mm Hg and 5 to 45 mm Hg. LC strains were analyzed for regional variations and the effect of LC area.

## Methods

The specimen preparation, SHG imaging, DVC algorithm, and strain calculation methods were described previously in Midgett et al.^9^ The following section briefly summarizes these methods and the methods used for RGC axonal loss grading, LC area calculation, and statistical analysis of the effects of glaucoma diagnosis, degree of axonal damage, and variations with LC region and area.

### Eye Tissues

Ten eyes from 6 donors with no prior history of glaucoma (Table 1) and 16 eyes from 8 donors diagnosed with glaucoma (Table 2) were obtained from the National Disease Research Interchange, Eversight, and the Minnesota Lions Eye Bank within 24 hours postmortem and subjected to inflation testing within 48 hours postmortem. All donors were of Caucasian descent and there were equal numbers of male and female donors. The normal and glaucoma groups had the same age range, 76 to 93 years, and a similar average age, 83*.*8 ± 6*.*1 years and 87*.*3 ± 5*.*4 years, respectively. Glaucoma eyes were confirmed based on retrieved medical records and/or confirmation of previous glaucoma diagnosis by family members. Five of eight glaucoma donors were diagnosed with POAG, one donor had chronic ACG, one donor had pseudoexfoliation glaucoma, and one had an unknown glaucoma type. Medical records for the 16 glaucoma eyes indicated the last IOP measurement for 14 eyes, the cup-to-disk ratio for 7 eyes, and visual field measurements for 6 eyes (Table 2).

**Table 1.** Donor Information for Eyes With no Glaucoma History

Eye ID	Age (y)	Sex	Side	Optic Nerve Damage
1	88	M	Left	ND
2^*^	90+	F	Left	NU
3^*^	90+	F	Right	NU
4^*^	79	M	Right	NU
5^*^	79	M	Left	NU
6	76	M	Right	NU
7^*^	83	F	Right	ND
8^*^	83	F	Left	NU
9^*^	84	F	Right	NU
10^*^	84	F	Left	NU

* Indicates left and right eyes from the same donor. NU indicates 10% or less optic axon loss; ND indicates >10% optic axon loss in masked, qualitative analysis of optic nerve thick sections embedded in epoxy.

**Table 2.** Donor Information for Eyes Diagnosed With Glaucoma

Eye	Age (y)	Sex	Side	Diagnosis	Last IOP	Visual Field	Cup-to-Disk	Optic Nerve Damage
11^*^	90+	M	Right	POAG	15	-9.37 dB	0.7	GS
12^*^	90+	M	Left	POAG	15	-6.29 dB	0.45	GM
13^*^	90+	F	Right	ACG	12	98% VFI	0.5	GM
14^*^	90+	F	Left	ACG	11	60% VFI	0.6	GS
15^*^	86	M	Right	Pseudoexfoliation	14	–	–	GS
16^*^^,^^†^	86	M	Left	Pseudoexfoliation	14	–	–	GM
17^*^^,^^†^	89	M	Right	Unknown	–	–	–	GM
18^*^^,^^†^	89	M	Left	Unknown	–	–	–	GM
19^*^	76	M	Right	POAG	22	–	–	GM
20^*^	76	M	Left	POAG	27	–	–	GM
21^*^	85	F	Right	POAG	13	–	–	GS
22^*^	85	F	Left	POAG	13	–	–	GS
23^*^	90+	F	Right	POAG	16	-1.8 dB	0.8	GM
24^*^	90+	F	Left	POAG	16	-3.1 dB	0.8	GM
25^*^	86	F	Right	POAG	13	–	0.5	GS
26^*^	86	F	Left	POAG	13	–	–	Unknown

* Indicates left and right eyes from the same donor.

† Indicates that the glaucoma diagnosis may be uncertain.

The categories GM indicate 25% or less optic axon loss and GS indicate >25% optic axon loss in masked, qualitative analysis of optic nerve thick sections embedded in epoxy.

Glaucoma diagnosis was provided by written material submitted by the institutions providing the postmortem eyes. In some cases, there was minimal information other than the diagnosis and the fact that typical glaucoma eye drop medication was used premortem. In other cases, some eye examination notes were available. To categorize the degree of axonal damage in glaucoma eyes, a masked, qualitative evaluation of axon loss in the optic nerve cross-sections in epoxy-embedded, thick sections was made. Optic nerve sections were excised from the eye for all specimens 1 to 3 mm posterior to the LC, fixed in a 4% paraformaldehyde solution, embedded in epoxy resin, and sectioned into 1-µm-thick slices. A glaucoma specialist (H.Q.), masked to the diagnosis, examined the sections, and assigned a grade for the degree of axon loss, as shown in prior studies^18^^–^^22^ (Table 1-2). The assigned grades were 10% or less loss, 10% to 25% loss, 25% to 50% loss, 50% to 75% loss, and 75% or more loss. In the normal group, the optic nerve of 8 of 10 eyes had an appearance of 10% or less loss, 1 had 10% to 25% loss, and 1 had 25% to 50% loss. Of the 16 eyes in the glaucoma group, the optic nerve of 6 eyes had an appearance of 10% or less loss, 3 had 10% to 25% loss, 3 had 25% to 50% loss, 3 had 50% to 75% loss, and 1 did not have enough optic nerve to obtain an adequate section for grading (unknown). No eyes had 75% or more loss. On masked regrading, the grading of three nerves changed between the 10% or less and the 10% to 25% loss categories. In this study, we have therefore divided the diagnosed glaucoma eyes to only two groups, those with 25% or less axon loss (GM) and those with greater than 25% axon (GS). The normal eyes are divided into undamaged (NU) with 10% or less axon loss and damaged with >10% damage. Eye 16 with pseudoexfoliation (PEX) glaucoma had 10% or less axon damage, whereas the PEX glaucoma eye 15 from the same donor had 25% to 50% axon damage. PEX glaucoma can often present unilaterally; thus, eye 16 may have been misdiagnosed as a glaucoma eye. Eyes 17 and 18 had an unknown glaucoma diagnosis. The history of glaucoma treatment of these eyes was equal to the other eyes, but the specific type of glaucoma was not determinable from the available clinical record. The optic nerves also had 10% or less axon damage, thus eyes 17 and 18 also may have been misdiagnosed as having glaucoma.

### Specimen Preparation

The eye length of eyes 4 through 26 was measured with calipers as the distance from the center of the cornea to the opposing posterior surface of the globe, just superior to where the optic nerve protruded. The extraocular tissues were removed from the donor eyes, and the optic nerve was excised 1 mm posterior to the scleral surface to avoid cutting into the LC. Multiple thin cuts were made to remove the myelinated posterior lamina tissue and expose the trabecular structure of the LC. The specimen was examined under a dissecting microscope after each cut to confirm the exposure of the LC. The eye was glued (Permabond 910, Electron Microscopy Sciences, Hatfield PA) to a custom polycarbonate ring 3 to 5 mm anterior to the equator, such that the ONH was centered in the ring. The cornea and anterior sclera were excised from the eye and the intraocular components, including the retina and choroid, were removed leaving only the posterior scleral shell. The posterior scleral specimen was kept hydrated in a 1 M phosphate-buffered saline throughout specimen preparation and inflation testing.

### Imaging

Posterior scleral cup specimens were mounted on a custom inflation chamber such that the posterior surface of the LC was aligned with the objective and imaging plane of a Zeiss 710 laser-scanning microscope (LSM 710 NLO, Zeiss, Inc., Oberkochen, Germany) as described previously in Midgett et al.^9^ Ocular pressure was set by a water column to a baseline pressure of 5 mm Hg and the specimen was allowed to equilibrate for at least 25 minutes before imaging to minimize the effects of tissue creep. Duplicate 2 × 2 tiled *z*-stacks were acquired back-to-back, with scans starting 300 µm below the posterior surface and taken at 5-µm intervals up to the cut surface of the LC, using a Chameleon Ultra II laser tuned to 780 nm. A 390 to 410 nm band pass filter was used to collect the backscattered SHG signal of the collagen structures of the LC using a 10 × 0.45 NA Apochromat objective with zoom factor set at 0.7 to 0.8× depending on the size of the LC. The tiles were imaged at 512 × 512 pixels and stitched with 15% overlap, which gave an in-plane resolution of 2.37 to 2.77 µm/pixel, depending on the zoom factor (Supplementary Table S1). Specimens were aligned such that the X direction in the images corresponded to the nasal-temporal direction, Y corresponded to the inferosuperior direction, and *Z* corresponded to the anteroposterior direction. Imaging was repeated at the additional pressures of 10 and 45 mm Hg.

The SHG image volumes were processed by an iterative deconvolution algorithm (Huygens Essentials, SVI, Hilversum, NL) to reduce noise and blur, and contrast was enhanced with contrast-limited piecewise adaptive histogram equalization^23^ in FIJI.^24^ The shape and area of the LC opening was estimated by importing the z-stack at 5 mm Hg for each eye into FIJI and calculating the maximum intensity projection of the SHG volume. The boundary between the LC and oversaturated peripapillary sclera region was defined by picking points manually on the maximum intensity projection. An ellipse was fit to the points using the Matlab function *fit_ellipse* (Ohad Gal, 2003) and used to segment the LC and sclera. The LC area was estimated as *A* = *πab*, where *a* and *b* were the calculated major and minor axes of the ellipse.

### Displacement and Strain Calculations

The Fast-Fourier Iterative DVC algorithm^25^ was applied to the enhanced SHG volumes to calculate the three-dimensional (3D) displacement of the imaged collagen structures within the LC between 5 to 10 mm Hg and 10 to 45 mm Hg every 4 × 4 × 2 pixels in *X*, *Y*, and *Z*.^9^ This corresponds to a displacement calculation every 8 to 10 µm in *X* and *Y* and every 10 µm in *Z*. The displacement fields between 5 to 10 and 10 to 45 mm Hg were used to obtain the cumulative displacement between 5 and 45 mm Hg.^9^

The displacement components, *U_X_*, *U_Y_*, and *U_Z_* were smoothed locally using a Gaussian filter and fit to polynomials in the *X, Y, Z* directions as shown in Midgett et al.^9^ The components of the 3D Green-Lagrange strain tensor in the *X −Y* plane were evaluated from the gradients of the fit displacement fields *U_X_, U_Y_, U_Z_* at each grid point *X, Y, Z* in the posterior LC volume as,

(1) $$E_{XX}=\frac{\partial U_{X}}{\partial X}+\frac{1}{2}\left({\left(\frac{\partial U_{X}}{\partial X}\right)}^{2}+{\left(\frac{\partial U_{Y}}{\partial X}\right)}^{2}+{\left(\frac{\partial U_{Z}}{\partial X}\right)}^{2}\right)$$

(2) $$E_{YY}=\frac{\partial U_{Y}}{\partial Y}+\frac{1}{2}\left({\left(\frac{\partial U_{Y}}{\partial Y}\right)}^{2}+{\left(\frac{\partial U_{Y}}{\partial Y}\right)}^{2}+{\left(\frac{\partial U_{Z}}{\partial Y}\right)}^{2}\right)$$

(3) $$E_{XY}=\frac{1}{2}\left(\frac{\partial U_{X}}{\partial Y}+\frac{\partial U_{Y}}{\partial X}+\frac{\partial U_{X}}{\partial X}\frac{\partial U_{X}}{\partial Y}+\frac{\partial U_{Y}}{\partial X}\frac{\partial U_{Y}}{\partial Y}+\frac{\partial U_{Z}}{\partial X}\frac{\partial U_{Z}}{\partial Y}\right).$$

The normal strain components *E_XX_* and *E_YY_* describe the tensile (positive) or compressive (negative) strain in the nasal-temporal direction and inferosuperior direction respectively, whereas the shear strain EXY describes the angle distortion between the *X* and *Y* directions. We also calculated the out-of-plane strain components, i.e., EZZ, EXZ, EYZ; however, displacements and strains in the *Z* direction exhibited larger DVC errors and are not reported here (Supplementary Section S1.2).

This analysis differs from a two-dimension DIC approach by taking into account the full 3D displacement field in calculating the in-plane Lagrangian strains (Equations. 1-3). The posterior displacement component *U_Z_* describes the posterior bulging of the LC caused by a pressure increase. Including the gradient of *U_Z_* in the strain calculation is needed to account for the contribution of the bulging deformation on the in-plane strains.

The strain components were used to calculate the maximum principal strain *E_max_* and the maximum shear strain Γ_*max*_ in the *X −Y* plane, which denote the tensile strain and shear strain, respectively, along the directions in which they are maximum:

(4) $$E_{\max}=\frac{E_{XX}+E_{YY}}{2}+\sqrt{{\left(\frac{E_{XX}-E_{YY}}{2}\right)}^{2}+E_{XY}^{2}}$$

(5) $$\Gamma_{\max}=\sqrt{{\left(\frac{E_{XX}-E_{YY}}{2}\right)}^{2}+E_{XY}^{2}}$$

A coordinate transformation was applied to calculate the strain components in cylindrical coordinates, which is more consistent with the cylindrical symmetry of the LC about the central retinal artery and vein (CRAV). The orientation angle *θ* for a given point in the LC was calculated as the angle between a line connecting the point and the CRAV center, which was manually selected.^9^ This orientation angle was used to transform the strain components as follows:

(6) $$E_{rr}=E_{XX}\cos^{2}\theta +2E_{XY}\cos\theta \sin\theta +E_{YY}\sin^{2}\theta$$

(7) $$E_{\theta \theta }=E_{XX}\sin^{2}\theta -2E_{XY}\cos\theta \sin\theta +E_{YY}\cos^{2}\theta$$

(8) $$E_{r\theta }=\left(E_{YY}-E_{XX}\right)\cos\theta \sin\theta -E_{XY}\left(\sin^{2}\theta -\cos^{2}\theta \right),$$

where *E_rr_* and *E_θθ_* denote the radial and circumferential strain of the LC. The posterior bowing and in-plane expansion of the LC both contribute to *E_rr_*, whereas *E_θθ_* arises only from the radial expansion of the LC.

The baseline positional errors and the DVC displacement and strain errors were estimated for each specimen (Supplementary Section S1) as shown previously.^9^ The baseline positional error, which includes factors such as creep, was estimated for all specimens by correlating the two duplicate image sets acquired at 5 mm Hg with DVC and summarized as the average and average magnitude of the displacements *U_X_, U_Y_, U_Z_* (Supplementary Table S1). DVC correlation errors were estimated by applying a numerical displacement and stretch to one of the duplicate image volumes at 5 mm Hg and correlating it with DVC to the second, undeformed image volume. DVC errors were summarized as the average and average absolute difference between the DVC calculations and the numerically applied displacements and strains (Supplementary Tables S2-S3). DVC displacement error fields and the DVC correlation coefficient were used to mask regions within the image volume that were dark or had *X* and *Y* displacement errors greater than 2 µm as previously described.^9^ Displacement and strain errors in the LC were averaged through all 26 eyes to obtain an estimate of the average DVC resolution within a typical LC. The average displacement error was less than 0.8 *µ*m for *U_X_,* 0.6 *µ*m for *U_Y_,* and 3.6 *µ*m for *U_Z_.* Average strain errors were less than 0.28% for *E_XX_,* 0.25% for *E_YY_,* and 0.16% for *E_XY_.*

### Statistical Analysis

**Figure 1.**
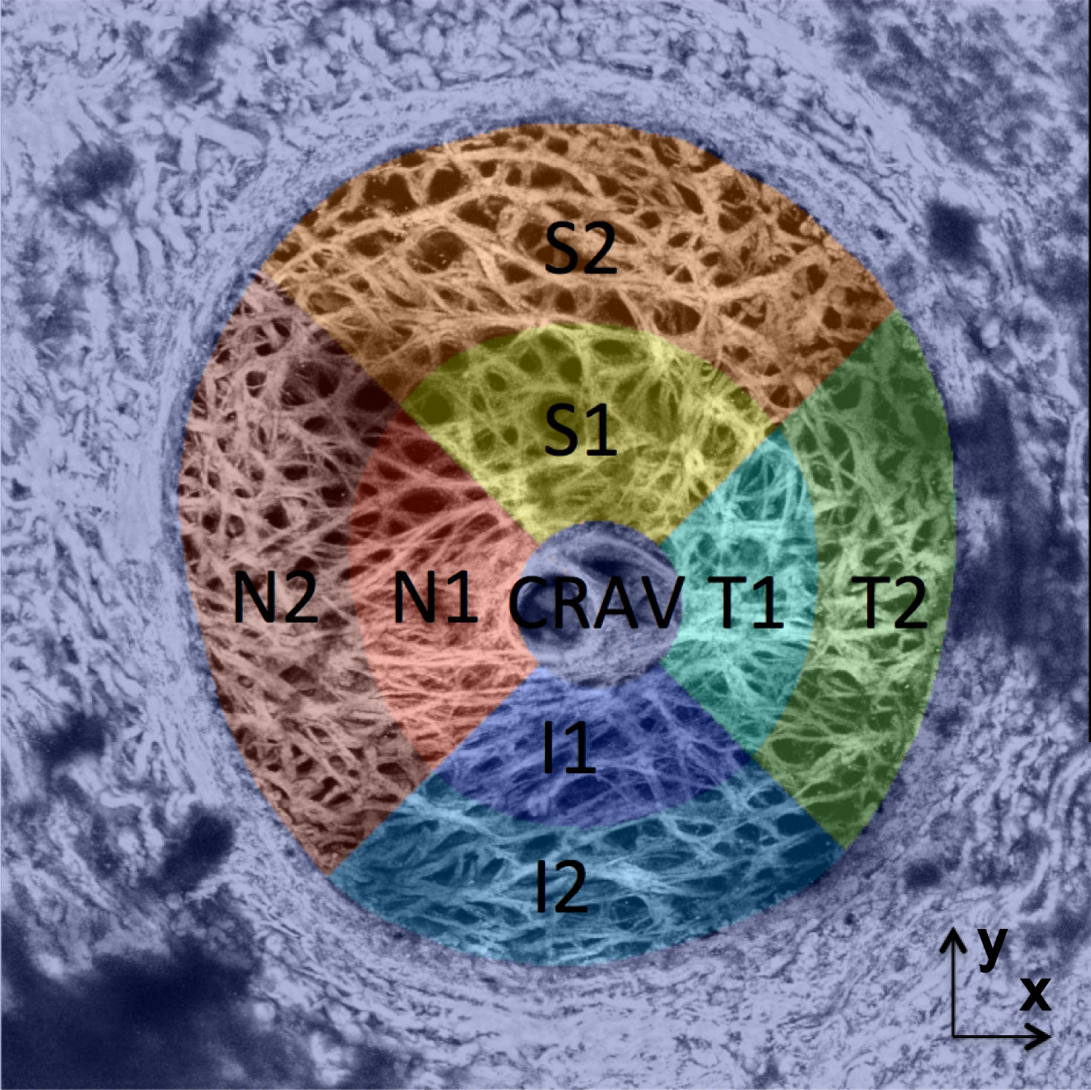
Illustration of the segmentation of the LC from the PPS and the 8 LC regions studied.

The LC was divided into eight anatomical regions as described previously.^11^ The center of the central retinal artery and vein (CRAV) was picked manually on the maximum intensity projection of the SHG image and a cylindrical region of radius 200 µm was defined surrounding the CRAV. The central (1) and peripheral (2) LC regions were divided at the midpoint distance between the LC boundary and the boundary of the CRAV region. The central and peripheral regions were further divided into the superior (S), inferior (I), temporal (T), and nasal (N) quadrants using 45° and 135° bisectors as shown in Figure 1.

The strain measures *E_XX_,* *E_YY_,* *E_rr_*, *E_θθ_,* *E_max_*, and Γ*_max_* between 5 to 10 and 5 to 45 mm Hg were averaged over the LC in each eye and in the eight regions of the LC. General linear models were used to test for: (1) associations between age and LC strain; (2) differences in eye length, LC area, and average LC strains between glaucoma and normal eyes; (3) differences in average LC strains between eye pairs from the same donor with the same or different levels of axonal loss; (4) differences in average LC strain between the undamaged normal group (NU, n = 8), the more mildly damaged glaucoma group (GM, n = 9), and more severely damaged glaucoma group (GS, n = 6); (5) differences between central and peripheral LC strains in the NU, GM, and GS groups; and (6) differences between the central nasal, temporal, superior, and inferior quadrant strains in the NU, GM, and GS groups. When comparing between glaucoma and normal eyes and between NU, GM, and GS groups, the analysis was performed for both including and excluding the uncertain glaucoma eyes listed in Table 2. In eye 12, the 2 × 2 tiled *z*-stacks acquired at 45 mm Hg failed to stitch together, so this eye was excluded in the analysis of strains between 5 and 45 mm Hg. In eye 26, the optic nerve damage was ungradable, so this eye was excluded from comparisons grouping eyes by level of axonal damage (NU, GM, GS). Eye length measurements were not taken for the first three eyes tested (1-3), so these eyes are excluded from comparisons of eye length. The peripheral LC quadrants were not compared because eyes with glaucoma damage often had poor peripheral correlation with one or more missing quadrants.

For analyses with one measurement per eye, such as the specimen-averaged strain outcomes, LC area, and eye length, all estimates and *p* values are from general linear models, which take into account correlations between the two eyes of a single donor. For all outcomes, the normal distribution function and the link identity function were used with the linear models. For analysis of data with more than one measurement per eye, all estimates and *p* values are from linear mixed models which take into account the clustering of the two eyes for a donor as well as correlations among the measurements from a single eye. Measurements from different LC regions were assumed to have a compound symmetry correlation structure, in which the measurements from any two regions have the same correlation. In the text, means and standard deviations are both estimated from the raw data. All *p* values are from regression models and least squares means from the models were used to estimate mean outcomes and 95% confidence intervals. The Bonferroni method was used to adjust significance levels for multiple pairwise comparisons of a dependent variable, such as in the analysis for the differences in the strain outcomes between the three categories of axon loss and the four quadrants. A comparison was considered significant if the *p* value (or adjusted *p* value, where applicable) was less than or equal to 0.05. All analyses were performed using SAS 9.2 (SAS Institute, Cary, NC).

## Results

### LC Geometry

**Figure 2.**
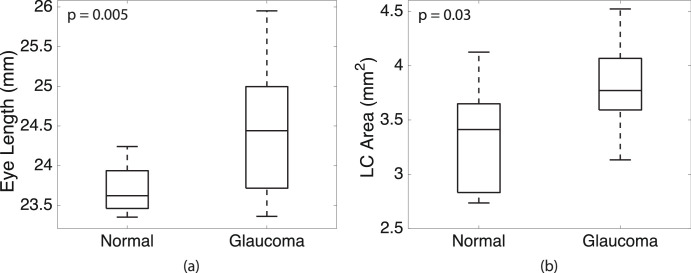
Comparison of (**A**) eye length (*p* = 0*.*005) and (**B**) LC area (*P* = 0*.*03) in eyes diagnosed with glaucoma (*n* = 16) and normals.

Eye length in the normal group (23.7 ± 0.3 mm, n = 7) was significantly smaller (*P* = 0*.*005, Table 3, Fig. 2a) than eye length in the glaucoma group (24.5 ± 0.8 mm, *n* = 16). LC area in the normal group (3.37 ± 0.51 mm^2^, *n* = 10) was also significantly smaller (*P* = 0*.*03, Table 3, Fig. 2b) than LC area of the glaucoma group (3.80 ± 0.37 mm^2^, *n* = 16). Eye length and LC area did not vary significantly between the GM and GS groups and was larger in both groups on average compared with normals (Table 4).

**Table 3.** Comparison of Eye Length and LC Area Between Normal (*n* = 10) and Glaucoma (*n* = 16) Groups

Outcome	Group	Number of Eyes	Estimated Mean Outcome (95% CI)	*P* Value
Eye length (mm)	Glaucoma	16	24.47 (23.98–24.96)	**0.005**
	Normal	7^*^	23.74 (23.59–23.88)	
LC area (mm2)	Glaucoma	16	3.81 (3.60–4.02)	**0.03**
	Normal	10	3.35 (2.98–3.72)	

Eye length and LC area were significantly larger (*P ≤* 0*.*03) in eyes diagnosed with glaucoma compared to normals.

* Eye length was not measured for the first three eyes tested (*n* = 7 for eye length and n = 10 for LC area). Bold numbers denote *P* values <= 0.05.

**Table 4.** Comparison of Eye Length and LC Area Between NU (*n* = 8), GM (*n* = 9), and GS (*n* = 6) Groups

Outcome	Group	Number of Eyes	Estimated Mean Outcome (95% CI)	Pairwise Comparison	*P* value	Adjusted *P* Value^*^
Eye Length (mm)	GS	6	24.42 (24.01–24.83)	GS - GM	0.80	1.00
	GM	9	24.45 (23.89–25.01)	GS - NU	**0.002**	**0.01**
	NU	6	23.72 (23.54–23.90)	GM - NU	**0.01**	**0.04**
LC Area (mm2)	GS	6	3.64 (3.40–3.88)	GS - GM	0.22	0.65
	GM	9	3.90 (3.58–4.21)	GS - NU	0.12	0.37
	NU	8	3.28 (2.89–3.67)	GM - NU	**0.02**	**0.05**

Eye length was significantly larger (*P* *≤* 0*.*04) in both GS and GM groups compared with the NU group and similar between the GM and GS groups. LC area was significantly larger (*P* = 0*.*05) in the GM group compared with the NU group, but there was no significant difference in LC area between the GS and NU groups or the GM and GS groups.

* *P* value adjusted for multiple comparisons. Bold numbers denote *P* values <= 0.05.

Excluding the three uncertain glaucoma eyes in Table 2 from the analyses changed the *p* values, but did not substantively alter the findings. The LC area (*P* = 0*.*03) and eye length (*P* = 0.02) remained smaller in the normal compared to glaucoma group. The LC area (*P* = 0.002) and eye length (*P* = 0.03) also differed between the different groups of axon loss. In post hoc pair-wise tests, the LC area was larger for the GM than NU group (adjusted *P* = 0.01) and larger in the GM than GS group (adjusted *P* = 0.004). The axial length was larger for the GS than NU group (adjusted *P* = 0.03)

### Strain Outcomes

**Figure 3.**
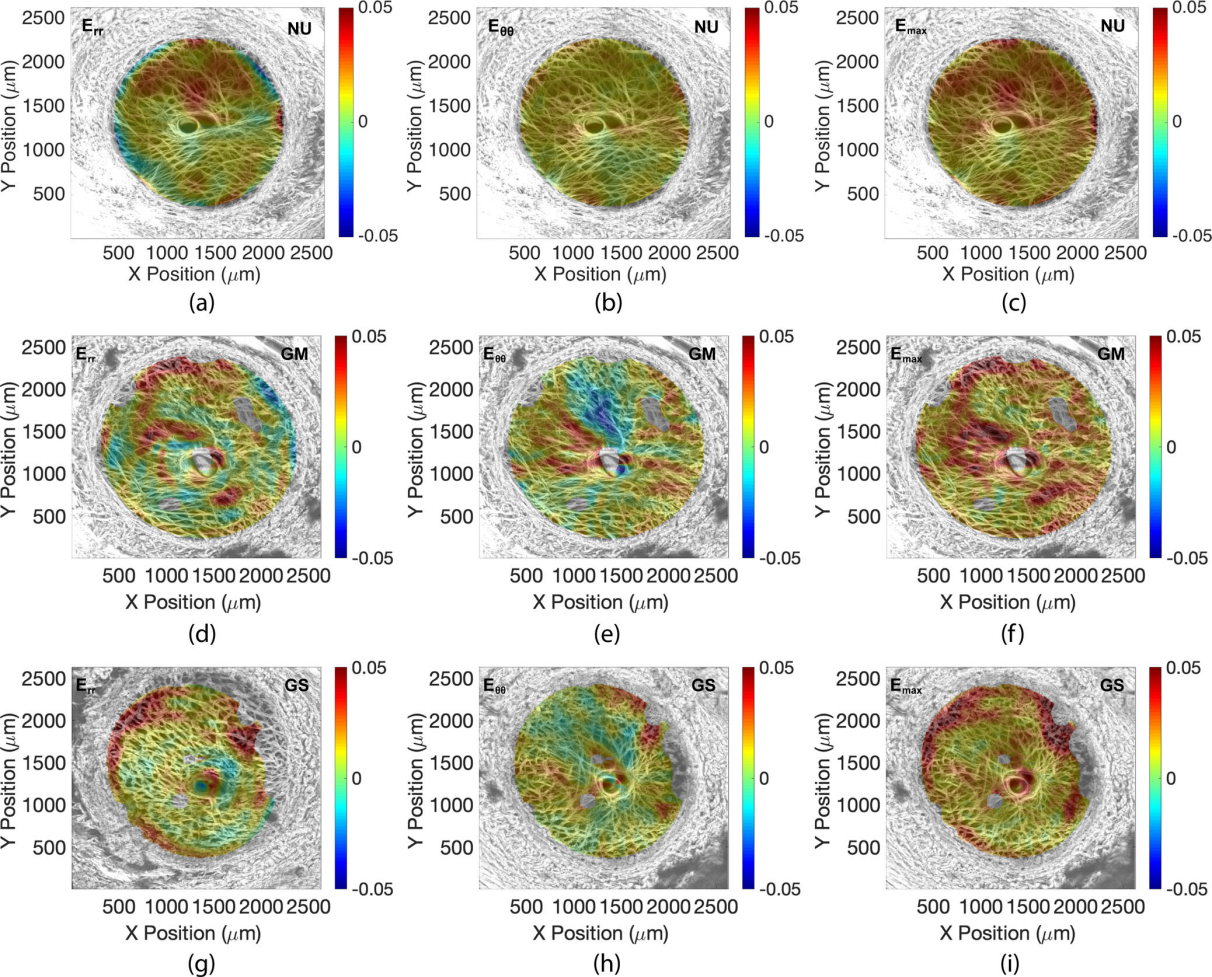
Comparison of strain within the LC of eye 6 (normal undamaged), eye 17 (glaucoma undamaged), and eye 14 (glaucoma severely damaged) for an inflation of 5 to 45 mm Hg. Eye 2: (**A**) *E_rr_*, (**B**) *E_θθ_*, (**C**) *E_max_*; eye 17: (**E**) *E_rr_*, (**E**) *E_θθ_*, (**F**) *E_max_*; and eye 14: (**G**) *E_rr_*, (**H**) *E_θθ_*, (**I**) *E_max_*. Holes in the strain color contours were regions that either had poor correlation coefficients or high displacement error estimates. These were removed from the strain calculations.

**Figure 4.**
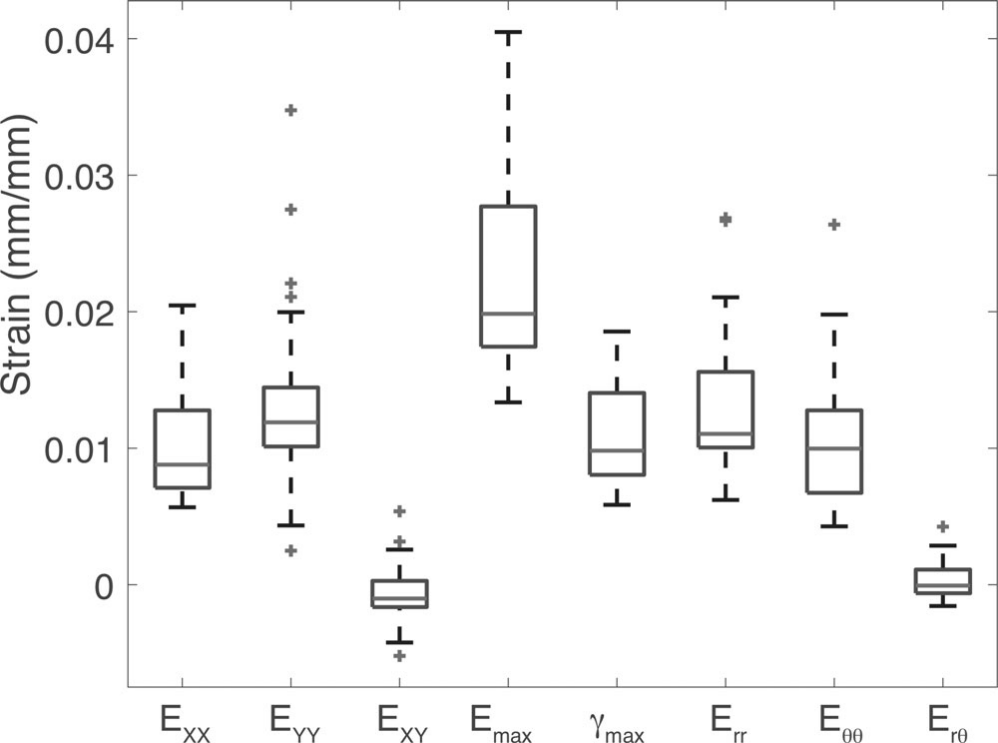
Comparison of strain outcomes of all specimens for inflation from 5 to 45 mm Hg (*n* = 25).

Contours of the strains *E_rr_*, *E_θθ_*, and *E_max_* are plotted in the LC for eye 6 (NU), eye 17 (GM), and eye 14 (GS) in Figure 3 and for all specimens in Supplementary Figures S2-S27. Compared across all specimens at 45 mm Hg (*n* = 25), maximum principal strain *E_max_* (2*.*30% ± 0*.*78%) was the largest of the LC strain outcomes. The normal strain components were of similar average magnitude (*E_XX_* = 1*.*02% ± 0*.*42%, *E_YY_* = 1*.*37% ± 0*.*70, *E_rr_* = 1*.*32% ± 0*.*57%, *E_θθ_* = 1*.*07% ± 0*.*55%) and were positive and significantly greater than zero (*P* *<* 0*.*0001, Table 5), showing that LC deformation from pressure increase was on average equibiaxial tension (Figure 4) in the plane of the tissue (LC expansion). The average shear strains *E_XY_* = *−*0*.*06% ± 0*.*23% and *E_rθ_* = 0*.*03% ± 0*.*14% were near-zero on average (*P* *>* 0*.*2, Table 5), but maximum shear strain was significant Γ*_max_* = 1*.*1% ± 0*.*39% (*P* *<* 0*.*0001, Table 5) because of local regions of large positive and negative shear strains.

**Figure 5.**
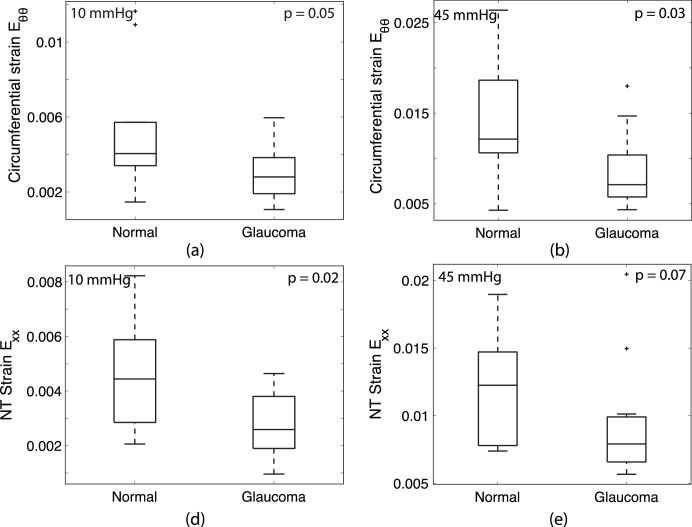
Comparison of average LC strain in normal and glaucoma eyes showing (**A**) *E_θθ_* at 10 mm Hg, (**B**) *E_θθ_* at 45 mm Hg, (**C**) *E_XX_* at 10 mm Hg, and (**D**) *E_XX_* at 45 mm Hg.

**Table 5.** Linear Models were Used to Estimate the Significance of Average Strain in all Eyes at 45 mm Hg (*n* = 25)

Strain Outcomes 45 mm Hg	Number of Measures	Estimated Strain (95% CI)	*P* Value
*E_XX_*	25	0.0102 (0.0085–0.0120)	**<0.0001**
*E_YY_*	25	0.0138 (0.0108–0.0167)	**<0.0001**
*E_XY_*	25	−0.0006 (−0.0015 to 0.0003)	0.21
*E_max_*	25	0.0230 (0.0200–0.0260)	**<0.0001**
Γ*_max_*	25	0.0110 (0.0096–0.0125)	**<0.0001**
*E_rr_*	25	0.0133 (0.0107–0.0158)	**<0.0001**
*E_θθ_*	25	0.0108 (0.0086–0.0130)	**<0.0001**
*E_rθ_*	25	0.0003 (−0.0003 to 0.0010)	0.32

Normal strain components were positive and significantly greater than zero (*P* *<* 0*.*0001), but shear strain components were near-zero on average (*P* *>* 0*.*3). Bold numbers denote *P* values <= 0.05.

#### Effects of Age

Average LC strains did not vary significantly with age between 5 and 10 or 5 and 45 mm Hg for the narrow and older age range (76-90+) of this study (*P* *≥* 0*.*08, Table 6, Supplementary Table S6-S7).

**Table 6.** Linear Models Were Used to Investigate the Variation of Strain With Age in all Eyes at 45 mm Hg (*n* = 25)

Strain Outcomes	Number of	Estimated Strain Change	
45 mm Hg	Measures	per 1 Year in Age (95% CI)	*P* Value
*E_XX_*	25	−0.000054 (−0.000379 to 0.000271)	0.74
*E_YY_*	25	0.000376 (−0.000049 to 0.000800)	0.08
*E_max_*	25	0.000223 (−0.000241 to 0.000686)	0.35
Γ*_max_*	25	0.000061 (−0.000177 to 0.000298)	0.62
*E_rr_*	25	0.000167 (−0.000235 to 0.000568)	0.42
*E_θθ_*	25	0.000156 (−0.000207 to 0.000520)	0.40

Strain measures did not vary significantly with age (*P* *≥* 0*.*08).

#### Effect of Glaucoma Diagnosis

The normal strain components and the maximum principal strain were generally smaller for diagnosed glaucoma eyes than for normal eyes at 10 and 45 mm Hg (Figure 5, Tables 7-8). At 10 mm Hg, *E_XX_* was 38% smaller (*P* = 0*.*02), *E_θθ_* was 46% smaller (*P* = 0*.*05), *E_max_* was 32% smaller (*P* = 0*.*08), and *E_rr_* was 36% smaller (*P* = 0*.*08) in glaucoma eyes (*n* = 16) compared with normals (*n* = 10) (Table 7). At 45 mm Hg, *E_θθ_* was 35% smaller (*P* = 0*.*03) and *E_XX_* was 25% smaller (*P* = 0*.*07) in glaucoma eyes (*n* = 15) compared with normals (*n* = 10) (Table 8).

**Table 7.** Comparison of LC Strain at 10 mm Hg Between Normal (*n* = 10) and Glaucoma (*n* = 16) Groups

Strain Outcomes 10 mm Hg	Group	Number of Eyes	Estimated Mean Outcome (95% CI)	*P* Value
*E_XX_*	Glaucoma	16	0.0028 (0.0022–0.0033)	**0.02**
	Normal	10	0.0045 (0.0032–0.0059)	
*E_YY_*	Glaucoma	16	0.0038 (0.0030–0.0047)	0.12
	Normal	10	0.0067 (0.0031–0.0103)	
*E_max_*	Glaucoma	16	0.0071 (0.0060–0.0082)	0.08
	Normal	10	0.0105 (0.0069–0.0140)	
Γ*_max_*	Glaucoma	16	0.0038 (0.0032–0.0043)	0.20
	Normal	10	0.0048 (0.0033–0.0064)	
*E_rr_*	Glaucoma	16	0.0037 (0.0031–0.0043)	0.08
	Normal	10	0.0058 (0.0036–0.0080)	
*E_θθ_*	Glaucoma	16	0.0029 (0.0022–0.0036)	**0.05**
	Normal	10	0.0054 (0.0030–0.0078)	

*E_XX_* and *E_θθ_* were significantly smaller (*P* *≤* 0*.*05) and *E_max_* and *E_rr_* were borderline significantly smaller (*P* = 0*.*08) in glaucomas compared with normals. Bold numbers denote *P* values <= 0.05.

**Table 8.** Comparison of LC Strain at 45 mm Hg Between Normal (*n* = 10) and Glaucoma (*n* = 15) Groups

Strain Outcomes 45 mm Hg	Group	Number of Eyes	Estimated Mean Outcome (95% CI)	*P* Value
*E_XX_*	Glaucoma	15	0.0090 (0.0070–0.0110)	0.07
	Normal	10	0.0120 (0.0095–0.0146)	
*E_YY_*	Glaucoma	15	0.0119 (0.0100–0.0137)	0.16
	Normal	10	0.0166 (0.0103–0.0230)	
*E_max_*	Glaucoma	15	0.0211 (0.0182–0.0240)	0.13
	Normal	10	0.0258 (0.0204–0.0313)	
Γ*_max_*	Glaucoma	15	0.0106 (0.0091–0.0122)	0.52
	Normal	10	0.0116 (0.0089–0.0144)	
*E_rr_*	Glaucoma	15	0.0121 (0.0097–0.0144)	0.30
	Normal	10	0.0149 (0.0102–0.0197)	
*E_θθ_*	Glaucoma	15	0.0088 (0.0071–0.0106)	**0.03**
	Normal	10	0.0136 (0.0096–0.0176)	

*E_θθ_* was significantly smaller (*P* = 0*.*03) and *E_XX_* was borderline significantly smaller (*P* = 0*.*07) in glaucomas compared with normals. Bold numbers denote *P* values <= 0.05.

Excluding the three uncertain glaucoma eyes in Table 2 caused the difference in the normal strains at 10 mm Hg between normal and diagnosed glaucoma eyes to become less significant for *E_XX_* (*P* = 0*.*03) but more significant for *E_θθ_* (*P* = 0*.*03), and *E_max_* (*P* = 0*.*06), and unchanged for *E_rr_*. Moreover, the difference in *E_YY_* became nearly significant *p* = 0*.*08. Similarly, at 45 mm Hg, the difference in *E_θθ_* became more significant (*P* = 0*.*02), but the difference in *E_XX_* became nonsignificant (*P* = 0*.*15).

#### Effect of Optic Nerve Damage

**Figure 6.**
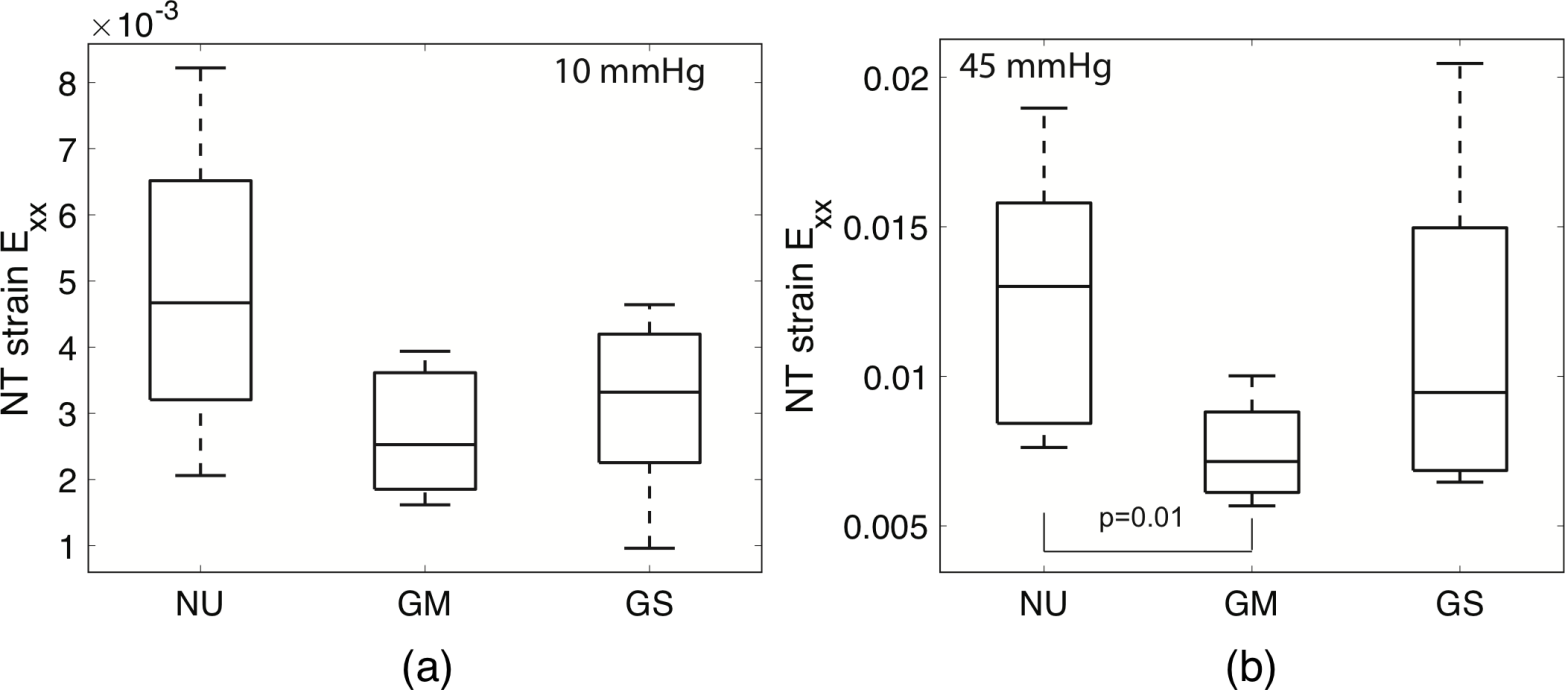
Comparison of *E_XX_* strain between undamaged normals (NU), more mildly damaged glaucomas (GM), and more severely damaged glaucomas (GS) at (**A**) 10 mm Hg and (**B**) 45 mm Hg. The number of specimens were NU: n = 8, GM: n = 9 for 10 mm Hg and n = 8 for 45 mm Hg), GS: n = 6. The *E_XX_* strain in glaucoma eyes was smaller but not significantly less than in normals at 10 mm Hg at all axon loss levels, but at 45 mm Hg, strain was only significantly smaller in undamaged or mildly-damaged glaucoma eyes.

We next compared the LC strains for the eyes separated into three groups depending on their glaucoma diagnosis and level of axon loss: undamaged normals (NU, n = 8), more mildly damaged glaucomas (GM, n = 9 for 10 mm Hg and n = 8 for 45 mm Hg), and more severely damaged glaucomas (GS, n = 6). For the normal (tensile) strain components and the maximum principal strain, the average for the GM and GS groups tended to be lower than the NU group. However, only *E_XX_* differed significantly between the different optic nerve damage groups (*P* = 0*.*003) at 45 mm Hg (Table 9). In a post hoc pairwise comparison, *E_XX_* was 40% smaller in the GM group compared to the NU group (adjusted *P* = 0*.*01) and 33% smaller than in the GS group, though the latter was not significant (adjusted *P* = 0*.*11, Fig. 6b). Though comparisons were not significant for the other strain outcomes, nearly all of the strain outcomes were smaller in the GM group than the NU group (Supplementary Tables S8-S9). At 45 mm Hg, all of the normal strain components in the^4^,^6^,^7^ GM group were also smaller than in the GS group (Supplementary Table S9).

**Table 9.** Comparison of *E_XX_* at 10 and 45 mm Hg Between the Normal Undamaged (NU), Glaucoma Undamaged and Mildly Damaged (GM) and Glaucoma Moderately and Severely Damaged (GS) Groups

Strain		Number of	Estimated Mean		Pairwise		Adjusted*
Outcome	Group	Eyes	Outcome (95% CI)	*P* Value	Comparison	*P* Value	*P* Value
*E_XX_*	GS	6	0.0028 (0.0018, 0.0038)		GS - GM	0.63	1.00
10 mm Hg	GM	9	0.0030 (0.0023, 0.0036)	0.11	GS - NU	**0.04**	0.11
	NU	8	0.0047 (0.0032, 0.0063)		GM - NU	**0.04**	0.13
*E_XX_*	GS	6	0.0113 (0.0080, 0.0146)		GS - GM	**0.04**	0.11
45 mm Hg	GM	8	0.0075 (0.0064, 0.0085)	**0.003**	GS - NU	0.58	1.00
	NU	8	0.0126 (0.0094, 0.0159)		GM - NU	**0.003**	**0.01**

Comparisons that were statistically significant are in bold.

**Figure 7.**
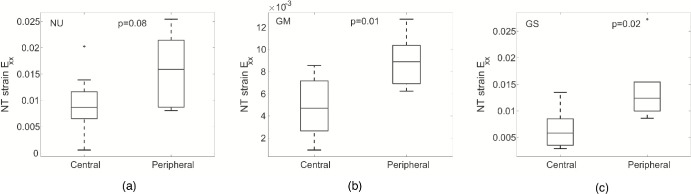
Comparison of *E_XX_* at 45 mm Hg in the central and peripheral LC for the (**A**) NU, (**B**) GM, and (**C**) GS groups.

Excluding the three uncertain glaucoma eyes reduced the number of eyes in the GM group to n = 6 at 10 mm Hg and n = 5 at 45 mm Hg, but it generally increased the significance of the comparisons (Table 10). At 10 mm Hg, both *E_XX_* and *E_YY_* were significantly different between the three groups (*P* *≤* 0*.*0001). In post hoc pairwise comparisons, *E_XX_* was significantly larger while *E_YY_* was significantly smaller in the more mildly damaged GM than more severely damaged GS glaucoma groups (adjusted *P* *≤* 0*.*0003) and *E_XX_* was nearly significantly larger in the NU than GS group (adjusted *P* = 0*.*07). The *p* values for *E_max_* and *E_θθ_* also decreased to *P* = 0*.*17, though they remained above the significance threshold. At 45 mm Hg, *E_XX_* remained significantly different between the groups, though with a higher *P* = 0*.*01, and larger for the NU than GM group (adjusted *P* = 0.03). In addition, the comparisons became significant for *E_θθ_* (*P* = 0*.*01), where *E_θθ_* was significantly larger for the NU than GM group (adjusted *P* = 0.02), and nearly significant for *E_max_* (*P* = 0*.*09).

**Table 10.** Comparison of *E_XX_ E_YY_* and *E_θθ_* at 10 and 45 mm Hg Between the Normal Undamaged (NU), more Mildly Damaged Glaucoma (GM) and more Severely Damaged Glaucoma (GS) Groups, Excluding the 3 Uncertain Glaucoma Eyes

Strain		Number of	Estimated Mean		Pairwise		Adjusted*
Outcome	Group	Eyes	Outcome (95% CI)	*P* value	Comparison	*P* value	*P* value
*E_XX_*	GS	6	0.0026 (0.0016–0.0036)		GS - GM	**<0.0001**	**<0.0003**
10 mm Hg	GM	6	0.0035 (0.0025–0.0045)	**<0.0001**	GS - NU	**0.02**	0.07
	NU	8	0.0047 (0.0032–0.0062)		GM - NU	0.18	0.55
*E_XX_*	GS	6	0.0113 (0.0080–0.0146)		GS - GM	0.07	0.21
45 mm Hg	GM	5	0.0079 (0.0066–0.0092)	**0.01**	GS - NU	0.57	1.00
	NU	8	0.0126 (0.0094–0.0159)		GM - NU	**0.01**	**0.03**
*E_YY_*	GS	6	0.0040 (0.0027–0.0052)		GS - GM	**<0.0001**	**<0.0003**
10 mm Hg	GM	6	0.0034 (0.0020–0.0047)	**0.0001**	GS - NU	0.37	1.00
	NU	8	0.0055 (0.0025–0.0085)		GM - NU	0.21	0.62
*E_YY_*	GS	6	0.0124 (0.0079–0.0169)		GS - GM	0.58	1.00
45 mm Hg	GM	5	0.0112 (0.0093–0.0130)	0.44	GS - NU	0.55	1.00
	NU	8	0.0146 (0.0092–0.0199)		GM - NU	0.24	0.71
*E_θθ_*	GS	6	0.0029 (0.0016–0.0041)		GS - GM	0.66	1.00
10 mm Hg	GM	6	0.0026 (0.0019–0.0034)	0.17	GS - NU	0.13	0.38
	NU	8	0.0048 (0.0026–0.0069)		GM - NU	0.06	0.19
*E_θθ_*	GS	6	0.0097 (0.0063–0.0132)		GS - GM	0.12	0.37
45 mm Hg	GM	5	0.0067 (0.0052–0.0082)	**0.01**	GS - NU	0.34	1.00
	NU	8	0.0122 (0.0086–0.0158)		GM - NU	**0.01**	**0.02**

The strains generally were smaller in the GM and GS groups compared with the NU group. Comparisons that were statistically significant are in bold.

#### Regional Strain Variation

The strain outcomes measured at 45 mm Hg were averaged within the central and^5^,^6^,^7^ peripheral LC regions for each damage group NU, GM, and GS to compare for differences in radial strain variation. In the NU group (*n* = 8), Γ*_max_* was significantly larger in the peripheral LC compared with central regions (*P* = 0*.*02) and *E_XX_* was borderline significantly larger in the peripheral LC region (*P* = 0*.*08) (Table 11, Figure 7a). In the GM group (*n* = 8), only *E_XX_* was significantly larger in the peripheral LC region compared to the central region (*P* = 0*.*01, Table 12, Figure 7b). In the GS group (*n* = 6), all of the normal strain components, *E_XX_*, *E_YY_*, *E_max_*, *E_rr_*, and *E_θθ_*, were all significantly larger in the peripheral LC region compared to the central LC region (*P* *≤* 0*.*05) and Γ*_max_* was borderline significantly larger in the peripheral LC region (*P* = 0*.*08) (Table 13, Figure 7c).

**Table 11.** Comparison of LC Strain in Central and Peripheral LC Regions of Undamaged Normal (NU) Eyes (*n* = 8)

NU Group Strain, 45 mm Hg	LC Location	Number of Measures	Estimated Mean Outcome (95% CI)	*P* Value
*E_XX_*	Central	8	0.0094 (0.0040–0.0147)	0.08
	Peripheral	8	0.0157 (0.0104–0.0211)	
*E_YY_*	Central	8	0.0152 (0.0080–0.0224)	0.93
	Peripheral	8	0.0150 (0.0077–0.0222)	
*E_max_*	Central	8	0.0214 (0.0127–0.0301)	0.10
	Peripheral	8	0.0290 (0.0202–0.0377)	
Γ*_max_*	Central	8	0.0091 (0.0042–0.0139)	**0.02**
	Peripheral	8	0.0136 (0.0087–0.0184)	
*E_rr_*	Central	8	0.0120 (0.0043–0.0197)	0.28
	Peripheral	8	0.0174 (0.0098–0.0251)	
*E_θθ_*	Central	8	0.0125 (0.0072–0.0177)	0.70
	Peripheral	8	0.0131 (0.0079–0.0184)	

Γ*_max_* was significantly higher in the peripheral LC region compared to the central region (*P* = 0*.*02). *E_XX_* was borderline significantly higher in the peripheral LC region compared to the central region (*P* *≤* 0*.*08).

**Table 12.** Comparison of LC Strain in Central and Peripheral LC Regions of More Mildly Damaged Glaucoma (GM) Eyes (*n* = 8)

GM Group Strain, 45 mm Hg	LC Location	Number of Measures	Estimated Mean Outcome (95% CI)	*P* value
*E_XX_*	Central	8	0.0048 (0.0028–0.0068)	**0.01**
	Peripheral	8	0.0089 (0.0069–0.0109)	
*E_YY_*	Central	8	0.0111 (0.0073–0.0149)	0.76
	Peripheral	8	0.0118 (0.0080–0.0157)	
*E_max_*	Central	8	0.0178 (0.0126–0.0230)	0.37
	Peripheral	8	0.0211 (0.0159–0.0263)	
Γ*_max_*	Central	8	0.0097 (0.0062–0.0133)	0.67
	Peripheral	8	0.0106 (0.0071–0.0142)	
*E_rr_*	Central	8	0.0083 (0.0051–0.0115)	0.12
	Peripheral	8	0.0121 (0.0089–0.0153)	
*E_θθ_*	Central	8	0.0077 (0.0042–0.0111)	0.38
	Peripheral	8	0.0088 (0.0053–0.0123)	

*E_XX_* was significantly higher in the peripheral LC region compared with the central region (*p* = 0*.*01).

**Table 13.** Comparison of LC Strain in Central and Peripheral LC Regions of More Severely Damaged Glaucoma (GS) Eyes (*n* = 6)

GS Group Strain, 45 mm Hg	LC Location	Number of Measures	Estimated Mean Outcome (95% CI)	*P* value
*E_XX_*	Central	6	0.0067 (0.0012–0.0122)	**0.02**
	Peripheral	6	0.0144 (0.0088–0.0199)	
*E_YY_*	Central	6	0.0088 (0.0013–0.0163)	**0.03**
	Peripheral	6	0.0154 (0.0079–0.0229)	
*E_max_*	Central	6	0.0154 (0.0051–0.0256)	**0.03**
	Peripheral	6	0.0273 (0.0170–0.0376)	
Γ*_max_*	Central	6	0.0076 (0.0032–0.0119)	0.08
	Peripheral	6	0.0124 (0.0081–0.0168)	
*E_rr_*	Central	6	0.0079 (0.0004–0.0154)	**0.01**
	Peripheral	6	0.0191 (0.0116–0.0266)	
*E_θθ_*	Central	6	0.0077 (0.0023–0.0130)	**0.02**
	Peripheral	6	0.0106 (0.0053–0.0160)	

*E_XX_*, *E_YY_*, *E_max_*, *E_rr_*, and *E_θθ_* were significantly higher in the peripheral LC region compared with the central region (*P ≤* 0*.*03).

The results suggested that the LC strain response for more severely damaged glaucoma eyes (GS) had the largest difference between central and peripheral. To test this hypothesis, we compared the ratio of the averaged peripheral to central maximum principal strain *E_max_* and maximum shear strain *γ*max between the three groups. The ratio of *E_max_* in the peripheral LC to central LC for inflation from 5 to 10 mm Hg was significantly different between the three groups (*P* = 0*.*01). The ratio was similar between the GM and NU (1.28 and 1.33), but was 20% larger in the GS (1.47) than GM group (adjusted *P* = 0*.*004). The ratio of peripheral to central LC strains were also larger in the GS than GM groups at 45 mm Hg, but the analysis was not statistically significant. The results did not substantively change when we excluded the three uncertain glaucoma eyes from the analysis. The difference in the ratio of *E_max_* in the peripheral to central LC at 10 mm Hg became more significant (*P* = 0*.*0001) as did the difference between GS and GM (adjusted *P* = 0*.*0003) in the *post-hoc* pairwise comparisons.

## Discussion

We applied the ex vivo inflation test developed by Midgett et al.^9^ to measure the strains in the LC of enucleated normal and glaucoma eyes caused by inflation from 5 to 10 mm Hg and 5 to 45 mm Hg. The strain outcomes were compared between normal and diagnosed glaucoma groups and between groups with different axonal damage levels. The specimen-averaged normal strains were smaller in the LC of diagnosed glaucoma eyes than in normal eyes. The comparisons were statistically significant for the nasal-temporal *E_XX_* and circumferential *E_θθ_* strains for inflation from 5 to 10 mm Hg and for *E_θθ_ for* inflation from 5 to 45 mm Hg. Excluding the three uncertain glaucoma eyes decreased the *p* values (making the results more significant) for *E_θθ_* and increased the *p* values for *E_XX_,* but did not otherwise alter the findings. The LC strains also differed between different damage groups. The average normal (tensile) strain components for the mildly damaged glaucoma GM and more severely damaged glaucoma GS groups tended to be smaller than for the undamaged normal NU group. At 45 mm Hg, the average normal strains also trended smaller for the GM than GS group. However, the comparison was only statistically significant for *E_XX_* at 45 mm Hg between the GM and NU groups. When we excluded the three uncertain glaucoma eyes, the comparison between the three groups became statistically significantly for more strain components, specifically for *E_XX_* and *E_YY_* at 10 mm Hg and *E_XX_* and *E_θθ_* at 45 mm Hg. Smaller strains indicate a stiffer structural response of the LC in glaucoma eyes compared to normal eyes. This is consistent with a number of previous findings for the eye, sclera and ONH tissues, including in vivo measurement of a stiffer ocular rigidity in glaucoma patients,^26^ measurements of a stiffer displacement response of the ONH in human postmortem eyes,^27^ a stiffer elastic modulus of postmortem monkey sclera with experimental glaucoma,^28^ a stiffer pressure-strain response of the human peripapillary sclera with glaucoma,^29^^–^^31^ and smaller anterior lamina displacement by in vivo ONH imaging with worse glaucoma damage.^32^

A number of factors can affect the strain outcomes measured for the inflation response of the LC, including age, geometry of the eye, LC and sclera, and material properties of the LC and sclera. Previous studies showed a significant stiffening effect with age, where nearly all strain outcomes decreased with age for a broad age range of 26 to 90+ years.^9^^,^^33^ However, we did not find a significant correlation between strain and age for the narrower and older age range of 75 to 90+ used in this study. This indicated that age-related variations did not contribute to the smaller strains measured for the diagnosed glaucoma group than the normal group, nor to the differences in strains between the different axonal damage groups.

The eye length of glaucoma eyes was on average 3% longer than in normal eyes. Based on Laplace's law for a thin spherical shell, a larger eye length would result in higher IOP-induced tensile hoop stresses in the sclera and higher LC strains rather than the smaller LC strains measured here for glaucoma eyes. However, the postmortem measurements of the lengths of the enucleated eyes were not made at the test pressures, thus differences in eye lengths and their effects on the hoop stresses and measured LC strain response to inflation may have been underestimated or overestimated in this discussion. The average LC area was 14% larger in glaucoma than normal eyes, which is consistent with previous findings that a larger LC area is associated with greater glaucoma prevalence.^1^^,^^34^ We measured in a prior study of 10 normal human eyes with a similar range of LC area (2.6–4.1 mm^2^) but a broader age range (26–73 years) that LC strains increased with LC area.^33^ This was opposite of the finding in this study that glaucoma eyes with larger LC area exhibited smaller strains, indicating that LC strains would be even less in glaucoma eyes compared to normal eyes if the two groups had similar eye lengths and LC areas.

The larger structural stiffness of the LC in glaucoma eyes may be caused in part by glaucoma-related remodeling of the sclera. Previous studies of postmortem human eyes reported a stiffer inflation response of the sclera^29^^,^^30^ and an altered anisotropic collagen fiber structure in the peripapillary sclera of glaucoma eyes.^30^^,^^35^ Studies in animal models of glaucoma using similar methods have shown that the inflation response of the sclera becomes stiffer with glaucoma induced by long-term IOP elevation.^28^^,^^31^ The collagen structure of the peripapillary sclera also became less anisotropic in mouse models of glaucoma.^36^ Computational models have shown that increasing the scleral stiffness relative to the LC stiffness decreases the scleral expansion and increases the posterior displacement of the LC and vice versa. A smaller scleral canal expansion and greater posterior bowing would manifest in inflation tests as a smaller circumferential strain *E_θθ_* and larger radial strain *E_rr_* in the LC. However, all specimen-averaged normal strain components, including the *E_θθ_* and *E_rr_*, trended smaller in glaucoma eyes, which suggested that the LC of glaucoma eyes were also stiffer than those of normal eyes. The differences in LC strains were more statistically significant for inflation to 10 mm Hg than to 45 mm Hg, which suggested that the nonlinear shape of the pressure-strain relationship was different between glaucoma and normal eyes. Inflation studies have also found that the peripapillary sclera of glaucoma human eyes^29^ and experimental monkey eyes^28^ exhibited a stiffer strain response in the low pressure region and a smaller transition stretch marking the strain-stiffening portion of the J-shaped pressure-strain curve. Subsequent finite element modeling studies fit the material parameters of a distributed fiber stress-strain model to the displacement field of the inflation tests and showed that the parameters associated with the collagen crimp and the matrix stiffness tended to be stiffer in glaucoma eyes than normal eyes.^28^^,^^30^ Sigal and coworkers mapped the collagen crimp in the lamina and cribrosa and peripapillary sclera^37^^,^^38^ and showed that the collagen crimp decreased with increased IOP, contributing to the characteristic nonlinear J-shaped, strain-stiffening stress response.^39^ Our findings motivate further studies of the collagen crimp structure in normal and glaucoma eyes.

We found larger differences with glaucoma for the normal strains than shear strains. The specimen averaged *E_XY_* and *Erθ* were near zero for both normal and glaucoma eyes. Differences in the maximum shear strain *γ_max_* between normal and glaucoma eyes and between the different damage groups were not significant, and were smaller than for the normal strains and the maximum principal strains. For the normal strains, we also found larger and more significant differences with glaucoma for the nasal-temporal strain *E_XX_* than the inferior-superior strain *E_YY_* and for the circumferential strain *E_θθ_* than the radial strain *E_rr_*. That glaucoma affected *E_XX_* more significantly than *E_YY_* may indicate differences in the anisotropy of the LC beam structure or in the oval shape of the LC between normal and glaucoma eyes. Gloster^40^ measured a more oval optic cup for glaucoma eyes with visual field defects than for non-glaucoma eyes with full visual fields, and we previously reported larger ratios of *E_XX_* and *E_YY_* for more oval LCs. The greater differences with glaucoma measured for *E_θθ_* than *E_rr_* may have been produced by the combined effects of LC and scleral remodeling. Computational models have shown that an increase in the stiffness^41^ and collagen anisotropy^42^ of the peripapillary sclera decrease the scleral canal expansion and increase the posterior LC displacement, while an increase in the LC stiffness decreases the posterior LC displacement. Further studies are needed to investigate the combined effects of alterations in stiffness and anisotropy of the peripapillary sclera and the LC on the different strain outcomes.

The pressure-induced strain response of the LC is also influenced by the thickness and curvature of the LC. The LC of advanced glaucoma eyes with severe optic axon damage are thinner and have a more excavated (cupped) appearance than the LC of normal and early glaucoma eyes.^1^^,^^2^^,^^43^^–^^45^ The thinner and more curved LC of glaucoma eyes may explain in part why the LC strains in the more severely damage glaucoma group were larger than those in the more mildly damaged group and why larger differences were measured between peripheral and central LC strains in the more severely damaged glaucoma group. We plan in future studies to estimate the curvature of the LC from 3D reconstruction of the SHG image volumes. After inflation testing, the eyes were fixed and sectioned for more detailed morphological characterization of the collagen structure.^46^ These will be analyzed in future work to estimate the thickness of the LC of the inflation tested specimens. Moreover, we are currently developing computational models and an inverse finite element method to estimate the mechanical properties of the LC from the DVC strain fields.

The finding that the LC of more mildly damaged glaucoma eyes tended to be stiffer than for undamaged normal eyes offer two intriguing possibilities. The strain response of the LC of early glaucoma eyes may have been stiffer at baseline, and this may have contributed to the development of glaucomatous axonal damage. Beotra et al.^12^ reported significantly lower ONH strains in vivo in ocular hypertension subjects, who are at higher risk for glaucoma, than healthy subjects for IOP elevation to 30 mm Hg, but no difference with glaucoma subjects. Alternatively, the stiffer LC strain response to inflation may have been caused by remodeling of the connective tissue structure and mechanical behavior in early glaucoma. Burgoyne and coworkers have shown using various methods, including postmortem histology ^47^ and in vivo videography^48^ and OCT imaging,^49^ that the posterior displacement response of the ONH of experimental glaucoma monkey eyes became more compliant than the contralateral eye after a couple of weeks of chronic IOP elevation before returning to normal after 13 weeks.^48^ The authors attributed the initial more compliant response to damage of the connective tissue structure of the ONH. The return to a normal displacement response may have been caused by remodeling effects in the early experimental glaucoma monkey eyes, such as thickening of the LC^50^ and increased connective tissue volume fraction,^51^ that stiffen the LC. If the LC were to become more compliant in the initial stage of the human disease, it may have occurred far too early to be detected by the present cross-sectional study of postmortem eyes. Rather, we may have detected in the more mildly damaged glaucoma group the subsequent stiffening effects of remodeling followed by an increase again in compliance in the more severely damaged glaucoma group at a later stage of the disease caused by ONH excavation, LC thinning and widening. Histological studies of glaucomatous human ONH have found alterations in the configuration of elastin,^52^ reorganization of fibril-forming collagens,^53^ and accumulation of s-GAGs and collagen IV in spaces formerly occupied by axons.^54^^–^^57^ These material remodeling effects would lead to a higher density of collagen, thus result in a stiffer LC stress-strain behavior and smaller LC inflation strains,^58^^,^^59^ while LC bowing, thinning, and widening would promote a more structurally compliant response and larger LC inflation strains with advancing glaucoma damage.

We have described the limitations of the inflation method using SHG and DVC in Midgett et al.^9^ The more posterior position and more bowed shape of the LC in glaucoma compared with normal eyes made cutting the optic nerve to expose the LC more difficult. The optic nerve section was cut at least 1 mm behind the peripapillary sclera and multiple thin slices were cut from the exposed surface to reveal the LC beams. Despite these precautions, the specimen preparation may have removed part of the LC. However, this would be more likely to occur in the more posteriorly bowed LC of glaucoma eyes, yet we measured smaller strains in these eyes, not the larger strains expected if significant portions of the LC were removed. Moreover, the larger strains that occurred in the moderately to severely damaged glaucoma eyes than in undamaged to mildly damaged eyes, occurred in the peripheral region of the LC rather than the central region that may have been more affected by the specimen preparation. We relied on qualitative evaluation by a glaucoma expert (H.Q.) of the level of axonal damage in thick sections of the optic nerve of donor eyes. Upon reexamination, the grade of 3 of the nerves changed between the <10% and 10%-25% damage levels, thus we divided the nerves into 2 coarser axon damage levels, a <25% and a >25% axon damage level. Glaucoma eyes with <10% axon damage may have been early in the stage of the disease or misdiagnosed as glaucoma. Recently developed automated axon counting methods may be able to provide a finer grading of axon damage. Automated axon counting methods have been successfully benchmarked against manual axon counting for the optic nerve sections of monkey eyes^60^^,^^61^ and rodent eyes.^62^^,^^63^ For the present study, the quality of axon preservation for some of the human donor eyes, which were received 24 to 48 hours postmortem, were too poor for accurate axon counts by current automated methods. The sample size was small, with only six eyes in the more severe glaucoma damage group and six to nine eyes in the more mildly damage group, and further studies with a larger number of specimens are needed to confirm the results of a stiffer pressure-strain response in the LC of glaucoma eyes compared to normal eyes. The donor eyes also had different types of glaucoma diagnosis. The majority of the glaucoma donors were diagnosed with POAG, but one donor had ACG, one had pseudoexfoliation glaucoma, and one had an unknown type of glaucoma. The type of glaucoma may also have a strong influence on the LC structure and properties, and separating these effects would require a substantially larger number of specimens. However, further investigations are merited on the basis of these outcomes.

## Conclusions

We measured the ex vivo inflation response of the posterior scleral cup of human donors in the age range of 76 to 93 years with and without glaucoma and analyzed the pressure-induced LC strains for the effect of glaucoma diagnosis, level of optic nerve damage, and age. The main findings were:

• LC strains were on average smaller in diagnosed glaucoma eyes compared with age-matched normal eyes. The difference in LC strains between the normal and glaucoma groups was larger and more statistically significant for inflation to 10 mm Hg than to 45 mm Hg.
• The LC tensile strains tended to be smaller in the mildly damaged glaucoma group than the undamaged normal group. At 45 mm Hg, the tensile strains were also smaller in the mildly damaged glaucoma group than in the severely damaged group. The result was statistically significant for *E_XX_* and *E_θθ_.*
• The more severely damaged eyes had significantly larger peripheral LC strains compared with central LC strains compared to the more mildly damaged glaucoma eyes

These findings suggest that the structural stiffness of the LC was larger in glaucoma eyes than age-matched normal eyes, and was larger at 45 mm Hg in more mildly damaged glaucoma eyes compared to undamaged normal eyes and more severely damaged glaucoma eyes. Differences in the structural stiffness of the LC observed in this study may represent both the effects of baseline properties that contribute to axon loss and the effects of remodeling in glaucoma. The lower LC strains in early glaucoma eyes may indicate stiffer baseline properties or connective tissue remodeling in the early disease before significant axon loss. Higher LC strains and larger difference between peripheral and central LC strains in advanced glaucoma eyes may reflect LC thinning, widening, and increased bowing with glaucoma. These findings support the need for further investigations to confirm and quantify differences in the mechanical behavior of the LC with glaucoma, and to study how the LC structure and mechanical behavior are remodeled by glaucoma and how they contribute to the susceptibility and progression of the disease.

## Supplementary Material

Supplement 1

## References

[1] Quigley HA, Varma R, Tielsch JM, Katz J, Sommer A, Gilbert DL The relationship between optic disc area and open-angle glaucoma: the Baltimore Eye Survey. *J Glaucoma*. 1999; 8: 347–352. 10604292

[2] Quigley HA, Addicks EM, Green WR, Maumenee AE Optic nerve damage in human glaucoma. II. The site of injury and susceptibility to damage. *Arch Ophthalmol. (Chicago, Ill.* 1960*)* 1981; 99: 635–649. 10.1001/archopht.1981.03930010635009 6164357

[3] Boland MV, Quigley HA. Risk factors and open-angle glaucoma: classification and application. *J Glaucoma*. 2007; 16: 406–418. 17571004 10.1097/IJG.0b013e31806540a1

[4] Cristina Leske M Predictors of long-term progression in the early manifest glaucoma trial. *Ophthalmic Epidemiol*. 2007; 14: 166–172. 17628686 10.1016/j.ophtha.2007.03.016

[5] Hernandez RM The optic nerve head in glaucoma: role of astrocytes in tissue remodeling. *Prog Retin Eye Reg.*. 2000; 19: 297–321. 10.1016/s1350-9462(99)00017-8 10749379

[6] Sigal IA, Flanagan JG, Tertinegg I, Ross Ethier C Modeling individual-specific human optic nerve head biomechanics. Part II: influence of material properties. *Biomech Model Mechanobiol*. 2009; 8: 99–109. 18301933 10.1007/s10237-008-0119-0

[7] Quigley HA, Sanchez RM, Dunkelberger GR, L'Hernault NL, Baginski TA Chronic glaucoma selectively damages large optic nerve fibers. *Invest Ophthalmol Visual Sci*. 1987; 28: 913–920. 3583630

[8] Girard MJA, Beotra MR, Chin KS, et al. In vivo 3-dimensional strain mapping of the optic nerve head following intraocular pressure lowering by trabeculectomy. *Ophthalmology*. 2016; 123: 1190–1200. 26992836 10.1016/j.ophtha.2016.02.008

[9] Midgett DE, Pease ME, Jefferys JL, et al. The pressure-induced deformation response of the human lamina cribrosa: analysis of regional variations. *Acta biomaterialia*. 2017; 53: 123–139. 28108378 10.1016/j.actbio.2016.12.054 PMC6053916

[10] Tran H, Grimm J, Wang B, et al. Mapping in-vivo optic nerve head strains caused by intraocular and intracranial pressures. *Proc SPIE Int Soc Opt Eng*. 2017; 10067: 100670B. doi:10.1117/12.2257360. PMC5880553 29618852

[11] Midgett DE, Jefferys JL, Quigley HA, Nguyen TD The contribution of sulfated glycosaminoglycans to the inflation response of the human optic nerve head. *Invest Ophthalmol Visual Sci*. 2018; 59: 3144–3154. 30025126 10.1167/iovs.18-23845 PMC6018372

[12] Beotra MR, Wang X, Tun TA, et al. In vivo three-dimensional lamina cribrosa strains in healthy, ocular hypertensive, and glaucoma eyes following acute intraocular pressure elevation. *Invest Ophthalmol Visual Sci*. 2018; 59: 260–272. 29340640 10.1167/iovs.17-21982

[13] Behkam R, Kollech HG, Jana A, et al. Racioethnic differences in the biomechanical response of the lamina cribrosa. *Acta Biomat*. 2019; 88: 131–140. 10.1016/j.actbio.2019.02.028 PMC6440866 30797107

[14] Nguyen C, Midgett D, Kimball EC, et al. Measuring deformation in the mouse optic nerve head and peripapillary sclera. *Investig Opthalmology Vis Sci*. 2017; 58: 721. 10.1167/iovs.16-20620 PMC5295769 28146237

[15] Nguyen C, Midgett D, Kimball EC, et al. Age-related changes in quantitative strain for mouse astrocytic lamina cribrosa and peripapillary sclera estimated using laser scanning microscopy in an explant model. *Invest Ophthalmol Vis Sci.* 2018; 59: 5157–5166. 30372742 10.1167/iovs.18-25111 PMC6516562

[16] Coudrillier B, Campbell IC, Read AT, et al. Effects of peripapillary scleral stiffening on the deformation of the lamina cribrosa. *Investig Opthalmol Vis Sci*. 2016; 57: 2666. 10.1167/iovs.15-18193 PMC4874475 27183053

[17] Wang Bo, Tran H, Smith MA, et al. In-vivo effects of intraocular and intracranial pressures on the lamina cribrosa microstructure. *PLoS One*. 2017; 12: e0188302. 29161320 10.1371/journal.pone.0188302 PMC5697865

[18] Morrison JC, Johnson EC, Cepurna W, Jia L Understanding mechanisms of pressure-induced optic nerve damage. *Prog Retin Eye Res*. 2005; 24: 217–240. 15610974 10.1016/j.preteyeres.2004.08.003

[19] Jia L, Cepurna WO, Johnson EC, Morrison JC Patterns of intraocular pressure elevation after aqueous humor outflow obstruction in rats. *Invest Ophthalmol Vis Sci*. 2000; 41: 1380–1385. 10798653

[20] Levkovitch-Verbin H, Quigley HA, Martin KR, Valenta D, Baumrind LA, Pease ME Translimbal laser photocoagulation to the trabecular meshwork as a model of glaucoma in rats. *Invest Ophthalmol Vis Sci.* 2002; 43: 402–410. 11818384

[21] Quigley HA Examination of the retinal nerve fiber layer in the recognition of early glaucoma damage. *Trans Am Ophthalmol Soc*. 1986; 84: 920–966. 3109098 PMC1298755

[22] Quigley HA, Dunkelberger GR, Green WR Retinal ganglion cell atrophy correlated with automated perimetry in human eyes with glaucoma. *Am J Ophthalmol*. 1989; 107: 453–464. 2712129 10.1016/0002-9394(89)90488-1

[23] Zuiderveld K Graphics Gems IV. Academic Press Professional Inc., 525 B Street Suite 1900 San Diego. CA USA, 4 edition, 1994;474–485.

[24] Schindelin J, Arganda-Carreras I, Frise E, et al. Fiji: an open-source platform for biological-image analysis. *Nature methods*. 2012; 9: 676. 22743772 10.1038/nmeth.2019 PMC3855844

[25] Bar-Kochba E, Toyjanova J, Andrews E, Kim K-S, Franck C A fast iterative digital volume correlation algorithm for large deformations. *Exp. Mech*. 2015; 55: 261–274.

[26] Hommer A, FuchsjaÂ´lger-Mayrl G, Resch H, Vass C, Garhofer G, Schmetterer L Estimation of ocular rigidity based on measurement of pulse amplitude using pneumotonometry and fundus pulse using laser interferometry in glaucoma. *Investig Opthalmol Vis Sci*. 2008; 49: 4046. 10.1167/iovs.07-1342 18487379

[27] Zeimer RC, Ogura Y The relation between glaucomatous damage and optic nerve head mechanical compliance. *Arch Ophthalmol*. 1989; 107: 1232–1234. 2527025 10.1001/archopht.1989.01070020298042

[28] Girard MJA, Suh J-KF, Bottlang M, Burgoyne CF, Downs JC Biomechanical changes in the sclera of monkey eyes exposed to chronic IOP eevations. *Investig Opthalmol Vis Sci*. jul 2011; 52: 5656. 10.1167/iovs.10-6927 PMC3176060 21519033

[29] Coudrillier B, Tian J, Alexander S, Myers KM, Quigley HA, Nguyen TD Biomechanics of the Human Posterior Sclera: Age- and Glaucoma-Related Changes Measured Using Inflation Testing. *Investig Opthalmol Vis Sci*. 2012; 53: 1714. 10.1167/iovs.11-8009 PMC3630906 22395883

[30] Coudrillier B, Pijanka JK, Jefferys JL, et al. Glaucoma-related changes in the mechanical properties and collagen micro-architecture of the human sclera. *PLoS One*. 2015; 10: e0131396. 26161963 10.1371/journal.pone.0131396 PMC4498780

[31] Nguyen C, Cone FE, Nguyen TD, et al. Studies of scleral biomechanical behavior related to susceptibility for retinal ganglion cell loss in experimental mouse glaucoma. *Invest Ophthalmol Vis Sci.* 2013; 54: 1767–1780. 23404116 10.1167/iovs.12-10952 PMC3626517

[32] Quigley H, Arora K, Idrees S, et al. Biomechanical Responses of lamina cribrosa to intraocular pressure change assessed by optical coherence tomography in glaucoma eyes. *Invest Ophthalmol Vis Sci.* 2017; 58: 2566–2577. 28494490 10.1167/iovs.16-21321

[33] Ling YTT, Shi R, Midgett D, Jefferys JL, Quigley HA, Nguyen TD Characterizing the collagen network structure and pressure-induced strains of the human lamina cribrosa. * Invest Ophthalmol Vis Sci.* 2019; 60: 2406–2422. 31157833 10.1167/iovs.18-25863 PMC6545820

[34] Healey PR, Mitchell P The relationship between optic disc area and open-angle glaucoma. *J. Glaucoma*. 2000; 9: 203–204. 10782635

[35] Pijanka JK, Coudrillier B, Ziegler K, et al. Quantitative mapping of collagen fiber orientation in non-glaucoma and glaucoma posterior human sclerae. *Invest Ophthalmol Vis Sci.* aug 2012; 53: 5258–70. 22786908 10.1167/iovs.12-9705 PMC3416032

[36] Pijanka JK, Spang MT, Sorensen T, et al. Depth-dependent changes in collagen organization in the human peripapillary sclera. *PLoS One*. 2015; 10: e0118648. 25714753 10.1371/journal.pone.0118648 PMC4340934

[37] Jan NJ, Lathrop K, I A Collagen architecture of the posterior pole: high-resolution wide field of view visualization and analysis using polarized light microscopy. *Investig Ophthalmol Vis Sci*. 2017; 58: 735–744. 28146238 10.1167/iovs.16-20772 PMC5295768

[38] Jan NJ, Gomez C, Moed S, Voorhees AP, Schuman JS, Bilonick RA, Sigal IA Microstructural crimp of the lamina cribrosa and peripapillary sclera collagen fibers. *Investig Ophthalmol Vis Sci*. 2017; 58: 3378–3388. 28687851 10.1167/iovs.17-21811 PMC5501496

[39] Jan NJ, Sigal IA Collagen fiber recruitment: a microstructural basis for the nonlinear response of the posterior pole of the eye to increases in intraocular pressure. *Acta Biomater*. 2018; 72: 295–305. 29574185 10.1016/j.actbio.2018.03.026 PMC6071322

[40] Gloster J Vertical ovalness of glaucomatous cupping. *Br J Ophthalmol*. 1975; 59: 721–724. 1218184 10.1136/bjo.59.12.721 PMC1017442

[41] Sigal IA, Yang H, Roberts MD, Burgoyne CF, Crawford Downs J IOP-induced lamina cribrosa displacement and scleral canal expansion: an analysis of factor interactions using parameterized eye-specific models. *Investig Opthalmol Vis Sci*. 2011; 52: 1896. 10.1167/iovs.10-5500 PMC3101679 20881292

[42] Coudrillier B, Boote C, Quigley HA, Nguyen TD Scleral anisotropy and its effects on the mechanical response of the optic nerve head. *Biomech Model Mechanobiol*. 2013; 12: 941–963. 23188256 10.1007/s10237-012-0455-y PMC3615129

[43] Quigley HA Open-angle glaucoma. *New Engl J Med*. 1993; 328: 1097–1106. 8455668 10.1056/NEJM199304153281507

[44] Jonas JB, Fernández MC, Stürmer J Pattern of glaucomatous neuroretinal rim loss. *Ophthalmology*. 1993; 100: 63–68. 8433829 10.1016/s0161-6420(13)31694-7

[45] Kwon YH, Fingert JH, Kuehn MH, Alward WLM Primary open-angle glaucoma. *New Engl J Med*. 2009; 360: 1113–1124. 19279343 10.1056/NEJMra0804630 PMC3700399

[46] Pijanka JK, Markov PP, Midgett DE, et al. Quantification of collagen fiber structure using second harmonic generation imaging and two-dimensional discrete Fourier transform analysis: application to the human optic nerve head. *J Biophotonics*. 2018; 12: e201800376. 10.1002/jbio.201800376 PMC6506269 30578592

[47] Bellezza AJ, Rintalan CJ, Thompson HW, Crawford Downs J, Hart RT, Burgoyne CF Deformation of the lamina cribrosa and anterior scleral canal wall in early experimental glaucoma. *Invest Ophthalmol Vis Sci.* 2003; 44: 623–637. 12556392 10.1167/iovs.01-1282

[48] Burgoyne CF, Quigley HA, Thompson HW, Vitale S, Varma R Early changes in optic disc compliance and surface position in experimental glaucoma. *Ophthalmology*. 1995; 102: 1800–9. 9098280 10.1016/s0161-6420(95)30791-9

[49] Ivers KM, Yang H, Gardiner SK, et al. In vivo detection of laminar and peripapillary scleral hypercompliance in early monkey experimental glaucoma. *Investig Opthalmol Vis Sci*. 2016; 57: OCT388. 10.1167/iovs.15-18666 PMC4968772 27409498

[50] Yang H, Downs JC, Girkin C, et al. 3-D Histomorphometry of the normal and early glaucomatous monkey optic nerve head: lamina cribrosa and peripapillary scleral position and thickness. *Investig Opthalmol Vis Sci*. 2007; 48: 4597. 10.1167/iovs.07-0349 PMC2764532 17898283

[51] Reynaud J, Lockwood H, Gardiner SK, Williams G, Yang H, Burgoyne CF Lamina cribrosa microarchitecture in monkey early experimental glaucoma: global change. *Investig Opthalmol Vis Sci.*. 2016; 57: 3451–3469. 10.1167/iovs.16-19474 PMC4961064 27362781

[52] Hernandez MR, Andrzejewska WM, Neufeld AH Changes in the extracellular matrix of the human optic nerve head in primary open-angle glaucoma. *Am J Ophthalmol*. 1990; 109: 180–188. 2405683 10.1016/s0002-9394(14)75984-7

[53] Quigley HAA, Dorman-Pease ME, Brown AE Quantitative study of collagen and elastin of the optic nerve head and sclera in human and experimental monkey glaucoma. *Curr Eye Res*. 1991; 10: 877–888. 1790718 10.3109/02713689109013884

[54] Fukuchi T, Sawaguchi S, Hara H, Shirakashi M, Iwata K Extracellular matrix changes of the optic nerve lamina cribrosa in monkey eyes with experimentally chronic glaucoma. *Graefe's Arch Clin Exp Ophthalmol*. 1992; 230: 421–427. 1521806 10.1007/BF00175926

[55] Morrison JC, Dorman-Pease ME, Dunkelberger GR, Quigley HA Optic nerve head extracellular matrix in primary optic atrophy and experimental glaucoma. *Arch Ophthalmol*. 1990; 108: 1020–1024. 2369339 10.1001/archopht.1990.01070090122053

[56] Fukuchi T, Sawaguchi S, Yue BYJT, Iwata K, Hara H, Kaiya T Sulfated proteoglycans in the lamina cribrosa of normal monkey eyes and monkey eyes with laser-induced glaucoma. *Exp. Eye Res*. 1994; 58: 231–243. 8157116 10.1006/exer.1994.1012

[57] Knepper PA, Goossens W, Hvizd M, Palmberg PF Glycosaminoglycans of the human trabecular meshwork in primary open-angle glaucoma. *Investig Opthalmol Vis Sci*. 1996; 37: 1360–1367. 8641839

[58] Voorhees AP, Jan NJ, Sigal IA Effects of collagen microstructure and material properties on the deformation of the neural tissues of the lamina cribrosa. *Acta Biomat*. 2017; 58: 278–290. 10.1016/j.actbio.2017.05.042 PMC5537032 28528864

[59] Voorhees AP, Jan NJ, Austin ME, et al. Lamina cribrosa pore shape and size as predictors of neural tissue mechanical insult. *Invest Ophthalmol Vis Sci.* 2017; 58: 5336–5346. 29049736 10.1167/iovs.17-22015 PMC5649511

[60] Kerrison JB, Buchanan K, Rosenberg ML, et al. Quantification of optic nerve axon loss associated with a relative afferent pupillary defect in the monkey. *Arch Ophthalmol*. 2001; 119: 1333–1341. 11545640 10.1001/archopht.119.9.1333

[61] Reynaud J, Cull G, Wang L, et al. Automated quantification of optic nerve axons in primate glaucomatous and normal eyes–method and comparison to semi-automated manual quantification. *Invest Ophthalmol Vis Sci.* 2012; 53: 2951–2959. 22467571 10.1167/iovs.11-9274 PMC3382379

[62] Zarei K, Scheetz TE, Christopher M, et al. Automated axon counting in rodent optic nerve sections with AxonJ. *Sci Rep*. 2016; 6: 1–11. 27226405 10.1038/srep26559 PMC4881014

[63] Ritch MD, Hannon BG, Read TA, et al. AxoNet: an AI-based tool to count retinal ganglion cell axons. arXiv. 2019, https://arxiv.org/abs/1908.02919. 10.1038/s41598-020-64898-1 PMC7228952 32415269

