# Supplementary material for: The Effects of Glaucoma on the Pressure-Induced Strain Response of the Human Lamina Cribrosa
Source: Invest Ophthalmol Vis Sci. 2020 Apr 28;61(4):41. doi: 10.1167/iovs.61.4.41 (PMC7401932; doi:10.1167/iovs.61.4.41)
Supplement: Supplement 1 [file iovs-61-4-41_s001.pdf]

S1 DVC error and uncertainty

S1.1 Baseline positional error and uncertainty

To calculate the baseline positional error, DVC was applied to correlate the duplicate tiled z-stacks acquired at each pressure. Because the 2 image volumes were acquired back-to-back at nominally the same conditions, the displacement field should be zero everywhere, and any non-zero displacements obtained by DVC provides an estimate of the displacement error. The absolute displacement error for each displacement component $I = X, Y$ or Z at each point $j = 1...M$ was evaluated as,

$$e^b_{U_{Ij}} = \sqrt{U^2_{I_j}}, \quad j = 1...M, \tag{1}$$

where M is the number of points in the volume. In addition, we defined the bias and uncertainty for the positional correlation for each specimen as the mean and standard deviation of the displacement components. The baseline positional error includes the DVC correlation error and tissue creep during image acquisition. The positional error from creep can be observed as small subpixel misalignments of the tiles within images (Fig. S1). This is due to the z-stacks for each tile being acquired sequentially.

Table S1 lists the baseline positional error: absolute average error (e^b), bias(U), and uncertainty for DVC analysis of two image volumes acquired sequentially at the highest pressure of 45 mmHg. The specimens were imaged after equilibration for at least 25 minutes and using a 5 μs pixel dwell time. The baseline error, bias, and uncertainty varied between specimens based on the individual creep rates. Averaged over all 26 specimens, the absolute errors were 0.609±0.602 μm in X, 0.431±1.178 μm in Y, and 0.380±0.379 μm in Z, which were smaller than 1/4 of a pixel for all 3 directions. The baseline absolute error was higher in the scanning direction X, which had an average absolute error 41% larger than in Y. The baseline bias and uncertainty, averaged over the 26 specimens, was 0.456±0.679 μm in X, 0.336±1.229 μm in Y, and 0.308±0.423 μm in Z.

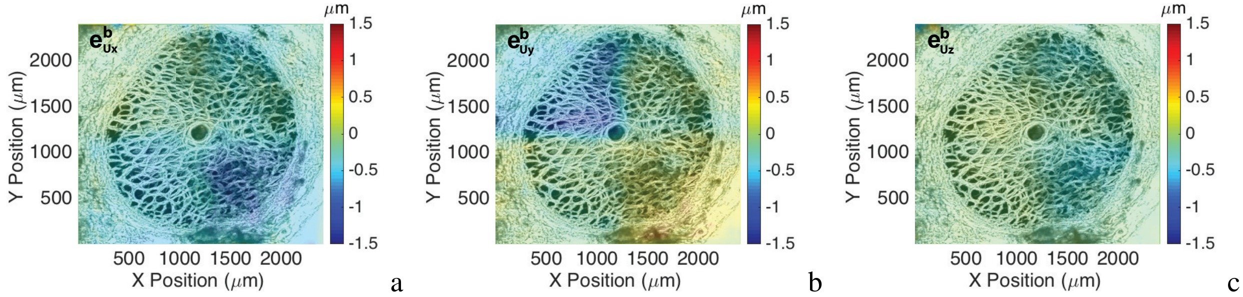

Figure S1: Thickness-averaged contour plots of the baseline positional errors a) $e^b_{U_X}$, b) $e^b_{U_Y}$, and c) $e^b_{U_Z}$, averaged through the thickness, for Specimen 2 at 45 mmHg after 30 minutes of equilibration. The maximum-intensity projection of the 2×2 tiled z-stacks is superimposed for illustrative purposes.

**Baseline Errors Eye ID**	**Diagnosis**	**X,Y Spacing** (μm)	**Z Spacing** (μm)	$e^b_{U_X}$ (μm)	$e^b_{U_Y}$ (μm)	$e^b_{U_Z}$ (μm)	U_X (μm)	U_Y (μm)	U_Z (μm)
1	Normal	2.37	3	0.328±0.334	0.265±0.299	0.327±0.534	-0.213±0.417	-0.225±0.330	-0.142±0.610
2	Normal	2.55	3	0.368±0.228	0.263±0.155	0.570±0.416	-0.355±0.249	-0.258±0.163	-0.542±0.452
3	Normal	2.55	3	0.420±0.261	0.310±0.281	0.219±0.211	0.394±0.300	0.157±0.388	0.133±0.273
4	Normal	2.77	3	0.551±0.613	0.215±0.306	0.290±0.327	-0.299±0.768	0.085±0.364	0.280±0.336
5	Normal	2.77	3	0.374±0.213	0.200±0.138	0.213±0.146	0.238±0.358	-0.174±0.170	0.208±0.153
6	Normal	2.77	5	1.117±0.276	0.173±0.139	0.427±0.378	-1.117±0.276	0.118±0.188	-0.419±0.386
7	Normal	2.77	5	0.530±0.434	0.158±0.215	0.117±0.145	-0.516±0.451	-0.013±0.267	-0.076±0.171
8	Normal	2.77	5	0.354±0.369	0.656±0.533	0.612±0.440	0.067±0.507	-0.642±0.550	0.602±0.453
9	Normal	2.77	5	0.282±0.241	0.196±0.184	0.093±0.093	-0.219±0.300	0.155±0.219	0.023±0.130
10	Normal	2.77	5	0.242±0.276	0.182±0.188	0.583±0.421	-0.020±0.367	-0.059±0.255	-0.572±0.437
11	Glaucoma	2.77	5	0.688±0.671	0.648±0.323	0.163±0.154	0.565±0.778	0.633±0.351	-0.073±0.211
12	Glaucoma	2.77	5	0.305±0.230	0.481±0.230	0.336±0.292	-0.254±0.285	-0.474±0.245	-0.315±0.315
13	Glaucoma	2.77	5	1.038±0.517	0.322±0.847	0.377±0.325	1.022±0.547	0.093±0.902	-0.335±0.369
14	Glaucoma	2.77	5	0.617±0.338	0.231±0.283	0.638±0.477	-0.586±0.389	0.076±0.357	-0.627±0.493
15	Glaucoma	2.77	5	0.960±0.922	0.750±0.463	0.320±0.324	0.698±1.133	0.729±0.495	0.134±0.436
16	Glaucoma	2.77	5	0.578±0.487	0.424±0.344	0.629±0.651	-0.079±0.752	-0.402±0.369	0.537±0.729
17	Glaucoma	2.77	5	1.327±0.791	0.623±0.389	0.545±0.470	1.312±0.815	0.601±0.422	0.523±0.495
18	Glaucoma	2.77	5	0.355±1.242	0.263±0.283	0.408±0.329	0.011±1.292	0.063±0.381	0.370±0.372
19	Glaucoma	2.77	5	0.300±0.327	0.387±0.335	0.310±0.243	0.212±0.390	-0.330±0.391	-0.283±0.273
20	Glaucoma	2.77	5	0.467±1.987	0.425±0.454	0.183±0.196	-0.340±2.013	-0.354±0.511	-0.062±0.261
21	Glaucoma	2.77	5	2.535±1.573	1.448±0.933	0.741±0.834	2.532±1.577	-1.438±0.949	0.729±0.845
22	Glaucoma	2.77	5	0.422±0.441	0.341±0.377	0.304±0.306	0.235±0.563	0.130±0.491	-0.216±0.373
23	Glaucoma	2.77	5	0.267±0.385	0.705±0.620	0.282±0.521	-0.019±0.468	0.591±0.729	-0.006±0.592
24	Glaucoma	2.77	5	0.284±0.635	0.557±21.397	0.262±0.450	0.023±0.695	0.048±21.404	-0.173±0.492
25	Glaucoma	2.77	5	0.862±0.488	0.492±0.426	0.554±0.579	-0.833±0.536	-0.383±0.525	0.508±0.620
26	Glaucoma	2.77	5	0.528±1.394	0.385±0.382	0.343±0.423	0.015±1.491	-0.305±0.449	0.109±0.533
Average	-	2.55, 2.77	3, 5	0.609±0.602	0.431±1.178	0.380±0.379	0.456±0.679	0.336±1.229	0.308±0.423

Table S1: The average and standard deviation of the absolute DVC baseline positional errors (e^b_U) and their bias (U) evaluated at 45 mmHg for the SHG images.

S1.2 Displacement error under uniform strain

We estimated the DVC displacement error under a uniform strain by numerically applying a rigid body displacement and a triaxial strain state to one of the duplicate tiled z-stacks taken at the 5 mmHg reference pressure using the Matlab R2015b functions *affine3d* and *imwarp*. The volumes were translated by 10 pixels in the X and Y directions, translated by 3 pixels (slices) in Z, stretched by 2% strain in the X and Y directions, and compressed by 5% strain along Z. These values corresponded to the typical average strains measured in the LC for pressurization from 5 mmHg to 10 mmHg. The FIDVC algorithm was applied to correlate the reference and numerically deformed SHG volumes for displacements. The DVC displacements were post-processed as described in Sec. 2.5 to remove poorly correlating regions and the strains were evaluated using eq.1 from the manuscript. The absolute error between the numerically applied displacement $U_{I_j}^{app}$ and strain $E_{IJ_j}^{app}$ components and the DVC displacement and strain components were evaluated at each point $j = 1..M$ as,

$$e_{U_{Ij}}^{c} = \sqrt{\left(U_{I_j}^{app} - U_{I_j}\right)^2}, \quad e_{E_{IJj}}^{c} = \sqrt{\left(E_{IJj}^{app} - E_{IJ_j}\right)^2}, \tag{2}$$

where $I, J = X, Y$ or Z. We also evaluated the bias and uncertainty in the DVC displacement and strain calculations from the mean and standard deviation of the difference between the applied and DVC fields.

Table S2 lists the absolute DVC displacement error and the displacement bias and uncertainty. Table S3 lists the absolute DVC strain error and the strain bias and uncertainty. The absolute displacement errors averaged over the 26 specimens were 0.785±0.414 μm in X, 0.535±0.343 μm in Y, and 3.507±2.301 μm in Z. While the displacement errors were greater than the baseline positional errors, they remained subpixel. The absolute strain errors averaged over the 26 specimens were 0.274±0.270% for E_{XX}, 0.235±0.245% for E_{YY}, 0.151±0.147% for E_{XY}, and 3.942±1.252% for E_{ZZ}. The bias and uncertainty in the displacements averaged over the 26 specimens were 0.673±0.505 μm in X, 0.377±0.466 μm in Y, and 1.117±3.908 μm in Z. The bias and uncertainty in the strain components averaged over 26 specimens were 0.037±0.382% for E_{XX}, 0.044±0.336% for E_{YY}, 0.016±0.209% for E_{XY}, and 3.845±1.426% for E_{ZZ}. The displacement bias was of the same order as the absolute error. In-plane strain bias, however, was an order of magnitude smaller than the absolute errors and smaller than 0.05% for all components.

Though the in-plane displacement errors were less than half a pixel, the out-of-plane displacement error was notably larger, approaching a pixel. Moreover, the average absolute out-of-plane strain E_{ZZ} error was 3.9%, which was nearly equivalent to the applied strain. High displacement and strain errors occurred in dark regions where DVC calculated zero displacement because of a lack of features. The in-plane displacement errors varied through the thickness and were largest in the most anterior and posterior 50 μm and were the smallest in the middle 200 μm of the 300 μm imaged volume. The out-of-plane displacement errors were near zero at the posterior surface and increased with Z-depth, likely because the image intensity and contrast worsened with depth.

**Correlation Errors Disp, Eye ID**	**Diagnosis**	$e^c_{U_X}$ (μm)	$e^c_{U_Y}$ (μm)	$e^c_{U_Z}$ (μm)	$U^{app}_X - U_X$ (μm)	$U^{app}_Y - U_Y$ (μm)	$U^{app}_Z - U_Z$ (μm)
1	Normal	0.612±0.295	0.491±0.257	1.777±1.427	0.599±0.320	-0.460±0.310	-0.196±2.271
2	Normal	0.506±0.369	0.323±0.226	2.871±2.049	-0.486±0.395	-0.136±0.370	-1.190±3.321
3	Normal	0.562±0.389	1.318±0.358	3.299±1.922	0.536±0.424	-1.315±0.371	-0.199±3.812
4	Normal	0.531±0.428	0.547±0.386	2.118±1.855	-0.201±0.652	0.409±0.530	-0.727±2.719
5	Normal	0.699±0.454	0.371±0.302	2.241±1.789	0.630±0.546	-0.064±0.474	-0.766±2.763
6	Normal	1.804±0.578	0.631±0.379	5.550±2.654	-1.802±0.583	-0.557±0.481	5.328±3.076
7	Normal	0.764±0.380	0.385±0.295	3.036±1.789	-0.755±0.397	-0.164±0.456	2.695±2.271
8	Normal	1.274±0.563	0.379±0.317	3.129±2.350	1.269±0.574	-0.037±0.493	0.631±3.862
9	Normal	1.429±0.373	0.411±0.300	3.224±1.762	-1.428±0.377	-0.320±0.395	0.756±3.596
10	Normal	0.854±0.580	0.465±0.338	3.734±2.135	0.699±0.759	-0.371±0.439	1.072±4.165
11	Glaucoma	0.443±0.332	0.338±0.314	4.045±3.193	-0.130±0.538	-0.034±0.460	-1.498±4.931
12	Glaucoma	0.311±0.256	0.357±0.239	3.039±1.975	-0.037±0.401	0.266±0.337	1.354±3.362
13	Glaucoma	1.244±0.452	0.503±0.358	4.399±2.450	-1.240±0.465	-0.088±0.611	1.098±4.914
14	Glaucoma	0.469±0.341	0.774±0.373	3.992±2.717	0.385±0.433	-0.702±0.495	0.436±4.809
15	Glaucoma	1.199±0.401	0.530±0.379	3.745±1.923	-1.193±0.417	-0.426±0.494	1.768±3.821
16	Glaucoma	0.980±0.414	0.590±0.382	3.741±2.065	0.954±0.470	-0.479±0.514	1.405±4.036
17	Glaucoma	0.767±0.482	0.325±0.292	3.524±2.443	-0.722±0.547	0.086±0.429	0.457±4.263
18	Glaucoma	0.616±0.424	0.351±0.316	3.897±3.100	0.568±0.486	0.186±0.434	-0.541±4.950
19	Glaucoma	0.971±0.368	0.438±0.296	4.071±3.065	-0.961±0.392	-0.313±0.426	-1.582±4.844
20	Glaucoma	0.533±0.427	0.433±0.382	3.991±2.715	0.289±0.619	-0.269±0.512	-0.332±4.815
21	Glaucoma	1.011±0.449	0.891±0.478	3.244±2.355	-0.996±0.481	-0.832±0.576	-2.653±3.004
22	Glaucoma	0.444±0.362	0.302±0.289	3.923±2.750	0.089±0.566	-0.005±0.418	-0.746±4.732
23	Glaucoma	0.459±0.385	0.344±0.290	3.430±2.162	-0.305±0.515	-0.095±0.440	0.205±4.050
24	Glaucoma	0.361±0.310	0.566±0.377	4.144±2.394	0.171±0.444	0.189±0.653	1.067±4.665
25	Glaucoma	0.981±0.452	0.895±0.523	3.332±2.303	-0.969±0.477	0.841±0.606	0.581±4.009
26	Glaucoma	0.580±0.417	0.828±0.411	3.841±2.487	0.188±0.689	-0.815±0.437	0.212±4.571
Average	-	0.785±0.414	0.535±0.343	3.507±2.301	0.673±0.505	0.377±0.466	1.117±3.908

Table S2: The average and standard deviation of the absolute DVC displacement errors (e^c_U) and their bias ($U^{app} - U$) evaluated at 45 mmHg for the SHG images.

Correlation Errors Strain, Eye ID	Diagnosis	$e^c_{E_{XX}}$ (μm)	$e^c_{E_{YY}}$ (μm)	$e^c_{E_{XY}}$ (μm)	$e^c_{E_{ZZ}}$ (μm)	$E^{app}_{XX} - E_{XX}$ (μm)	$E^{app}_{YY} - E_{YY}$ (μm)	$E^{app}_{XY} - E_{XY}$ (μm)	$E^{app}_{ZZ} - E_{ZZ}$ (μm)
1	Normal	0.108±0.159	0.093±0.157	0.075±0.097	4.034±1.012	-0.010±0.192	-0.037±0.178	0.012±0.122	4.030±1.028
2	Normal	0.092±0.115	0.089±0.097	0.061±0.068	4.367±1.136	-0.020±0.146	0.010±0.131	0.020±0.089	4.362±1.153
3	Normal	0.153±0.197	0.128±0.168	0.093±0.110	4.501±0.510	-0.017±0.249	-0.003±0.211	0.010±0.144	4.501±0.510
4	Normal	0.267±0.274	0.174±0.224	0.139±0.148	2.998±1.172	-0.028±0.381	-0.061±0.277	0.002±0.203	2.603±1.893
5	Normal	0.205±0.264	0.167±0.205	0.136±0.142	2.542±1.079	-0.007±0.334	0.006±0.265	0.009±0.197	1.833±2.065
6	Normal	0.262±0.311	0.215±0.259	0.180±0.190	4.288±1.109	0.034±0.405	0.005±0.336	0.011±0.261	4.273±1.167
7	Normal	0.117±0.143	0.121±0.181	0.083±0.102	1.206±0.951	0.012±0.185	-0.046±0.213	0.002±0.132	0.860±1.272
8	Normal	0.204±0.227	0.174±0.219	0.146±0.139	3.465±1.613	-0.012±0.305	-0.005±0.280	0.011±0.201	3.403±1.739
9	Normal	0.150±0.170	0.168±0.197	0.103±0.114	4.610±2.359	-0.019±0.226	-0.058±0.253	-0.007±0.153	4.560±2.455
10	Normal	0.192±0.209	0.156±0.198	0.149±0.153	4.382±1.228	0.007±0.284	-0.074±0.241	-0.069±0.202	4.356±1.317
11	Glaucoma	0.301±0.277	0.251±0.274	0.153±0.143	4.635±1.137	-0.055±0.405	-0.027±0.370	-0.004±0.210	4.635±1.137
12	Glaucoma	0.247±0.233	0.215±0.200	0.133±0.114	2.725±1.480	0.004±0.340	-0.006±0.293	-0.009±0.175	2.718±1.493
13	Glaucoma	0.317±0.288	0.321±0.306	0.178±0.168	4.943±1.293	-0.032±0.427	-0.109±0.430	-0.025±0.244	4.941±1.300
14	Glaucoma	0.296±0.272	0.311±0.301	0.174±0.170	4.601±1.182	-0.008±0.402	-0.032±0.431	-0.021±0.242	4.601±1.182
15	Glaucoma	0.294±0.290	0.305±0.289	0.171±0.160	3.568±1.397	-0.047±0.410	-0.037±0.419	-0.002±0.234	3.542±1.461
16	Glaucoma	0.406±0.346	0.396±0.351	0.205±0.185	3.898±1.247	-0.030±0.533	-0.089±0.521	0.002±0.276	3.898±1.247
17	Glaucoma	0.388±0.322	0.270±0.263	0.191±0.163	3.884±1.457	-0.040±0.503	0.019±0.376	-0.011±0.251	3.732±1.813
18	Glaucoma	0.384±0.336	0.267±0.260	0.180±0.148	4.649±1.464	-0.002±0.510	-0.011±0.373	-0.013±0.233	4.631±1.522
19	Glaucoma	0.294±0.247	0.266±0.274	0.144±0.130	4.776±1.003	-0.010±0.385	-0.015±0.381	0.000±0.194	4.776±1.003
20	Glaucoma	0.467±0.406	0.312±0.283	0.196±0.165	3.708±1.412	-0.096±0.611	-0.057±0.417	-0.016±0.255	3.645±1.568
21	Glaucoma	0.372±0.324	0.367±0.316	0.203±0.183	3.568±0.891	-0.116±0.479	-0.201±0.440	-0.076±0.263	3.537±1.004
22	Glaucoma	0.362±0.326	0.271±0.267	0.172±0.160	4.466±1.264	-0.032±0.486	-0.005±0.380	-0.027±0.233	4.378±1.541
23	Glaucoma	0.363±0.332	0.308±0.289	0.200±0.178	3.935±1.217	-0.123±0.477	-0.051±0.420	-0.013±0.268	3.869±1.411
24	Glaucoma	0.329±0.356	0.367±0.320	0.184±0.171	4.132±1.279	-0.036±0.483	-0.110±0.474	0.009±0.251	4.056±1.503
25	Glaucoma	0.227±0.236	0.236±0.278	0.145±0.154	4.307±1.150	-0.057±0.322	-0.050±0.361	0.026±0.209	4.168±1.581
26	Glaucoma	0.271±0.298	0.154±0.183	0.131±0.131	4.271±1.327	-0.054±0.399	-0.007±0.239	0.006±0.185	4.235±1.436
Average	-	0.274±0.270	0.235±0.245	0.151±0.147	3.942±1.252	0.037±0.382	0.044±0.336	0.016±0.209	3.845±1.426

Table S3: The average and standard deviation of the absolute DVC strain errors (e^c_E) and their bias ($E^{app} - E$) evaluated at 45 mmHg for the SHG images.

**Strain Outcomes 10 mmHg**	**Number of Measures**	**Estimated Strain (95% CI)**	**p-value**
E_{XX}	26	0.0035 (0.0027, 0.0043)	$<$ **0.0001**
E_{YY}	26	0.0050 (0.0033, 0.0067)	$<$ **0.0001**
E_{XY}	26	-0.0002 (-0.0005, 0.0001)	0.28
E_{max}	26	0.0085 (0.0067, 0.0103)	$<$ **0.0001**
Γ_{max}	26	0.0042 (0.0035, 0.0050)	$<$ **0.0001**
E_{rr}	26	0.0046 (0.0035, 0.0058)	$<$ **0.0001**
$E_{\theta\theta}$	26	0.0039 (0.0027, 0.0051)	$<$ **0.0001**
$E_{r\theta}$	26	0.0001 (-0.0002, 0.0003)	0.6

Table S4: Linear models were used to estimate the significance of average strain in all eyes at 45 mmHg ($n = 25$). Normal strain components were positive and significantly greater than zero ($p < 0.0001$), but shear strain components was near-zero on average ($p > 0.3$).

S2 Statistics

The strains E_{rr}, $E_{\theta\theta}$, and E_{max} are plotted for each eye in Figures S2-S27. Compared across all specimens at 45 mmHg, maximum principal strain E_{max} ($2.30\% \pm 0.78\%$) was the largest of the LC strain outcomes. The normal strain components were of similar average magnitude ($E_{XX} = 1.02\% \pm 0.42\%$, $E_{YY} = 1.37\% \pm 0.70$, $E_{rr} = 1.32\% \pm 0.57\%$, $E_{\theta\theta} = 1.07\% \pm 0.55\%$) and were positive and significantly greater than zero ($p < 0.0001, n = 25$, Table S5), showing that LC deformation from pressure increase was on average equibiaxial tension in the plane of the tissue (LC expansion). The average shear strains $E_{XY} = -0.06\% \pm 0.23\%$ and $E_{r\theta} = 0.03\% \pm 0.14\%$ were near-zero on average ($p > 0.2$, Table S5), but maximum shear strain was significant $\Gamma_{max} = 1.1\% \pm 0.39\%$ ($p < 0.0001, n = 25$, Table S5) because of local regions of large positive and negative shear strains.

Strain Outcomes 45 mmHg	Number of Measures	Estimated Strain (95% CI)	p-value
E_{XX}	25	0.0102 (0.0085, 0.0120)	$<$ **0.0001**
E_{YY}	25	0.0138 (0.0108, 0.0167)	$<$ **0.0001**
E_{XY}	25	-0.0006 (-0.0015, 0.0003)	0.21
E_{max}	25	0.0230 (0.0200, 0.0260)	$<$ **0.0001**
Γ_{max}	25	0.0110 (0.0096, 0.0125)	$<$ **0.0001**
E_{rr}	25	0.0133 (0.0107, 0.0158)	$<$ **0.0001**
$E_{\theta\theta}$	25	0.0108 (0.0086, 0.0130)	$<$ **0.0001**
$E_{r\theta}$	25	0.0003 (-0.0003, 0.0010)	0.32

Table S5: Linear models were used to estimate the significance of average strain in all eyes at 45 mmHg $(n = 25)$. Normal strain components were positive and significantly greater than zero ($p < 0.0001$), but shear strain components was near-zero on average ($p > 0.3$).

Strain Outcomes 10 mmHg	Number of Measures	Estimated strain change per 1 year in age (95% CI)	p-value
E_{XX}	26	-0.000017 (-0.000184, 0.000151)	0.85
E_{YY}	26	0.000193 (-0.000073, 0.000460)	0.16
E_{max}	26	0.000172 (-0.000140, 0.000484)	0.28
Γ_{max}	26	0.000081 (-0.000052, 0.000214)	0.23
E_{rr}	26	0.000057 (-0.000156, 0.000269)	0.6
$E_{\theta\theta}$	26	0.000122 (-0.000079, 0.000323)	0.23

Table S6: Linear models were used to investigate the variation of strain with age in all eyes at 45 mmHg $(n = 25)$. Strain measures did not vary significantly with age ($p > 0.1$).

Strain Outcomes 45 mmHg	Number of Measures	Estimated strain change per 1 year in age (95% CI)	p-value
E_{XX}	25	-0.000054 (-0.000379, 0.000271)	0.74
E_{YY}	25	0.000376 (-0.000049, 0.000800)	0.08
E_{max}	25	0.000223 (-0.000241, 0.000686)	0.35
Γ_{max}	25	0.000061 (-0.000177, 0.000298)	0.62
E_{rr}	25	0.000167 (-0.000235, 0.000568)	0.42
$E_{\theta\theta}$	25	0.000156 (-0.000207, 0.000520)	0.4

Table S7: Linear models were used to investigate the variation of strain with age in all eyes at 45 mmHg $(n = 25)$. Strain measures did not vary significantly with age ($p \geq 0.08$).

Strain Outcome 10 mmHg	Group	Number of Eyes	Estimated Mean Outcome (95% CI)	Pairwise Comparison	p-value	Adjusted* p-value
E_{XX}	GS	6	0.0028 (0.0018, 0.0038)	GS - GM	0.63	1.00
	GM	9	0.0030 (0.0023, 0.0036)	GS - NU	**0.04**	0.11
	NU	8	0.0047 (0.0032, 0.0063)	GM - NU	**0.04**	0.13
E_{YY}	GS	6	0.0039 (0.0024, 0.0053)	GS - GM	0.71	1.00
	GM	9	0.0042 (0.0029, 0.0054)	GS - NU	0.35	1.00
	NU	8	0.0055 (0.0024, 0.0086)	GM - NU	0.43	1.00
E_{max}	GS	6	0.0071 (0.0055, 0.0087)	GS - GM	0.41	1.00
	GM	9	0.0075 (0.0061, 0.0088)	GS - NU	0.15	0.46
	NU	8	0.0099 (0.0064, 0.0134)	GM - NU	0.20	0.59
Γ_{max}	GS	6	0.0038 (0.0031, 0.0046)	GS - GM	0.95	1.00
	GM	9	0.0038 (0.0032, 0.0044)	GS - NU	0.32	0.96
	NU	8	0.0048 (0.0030, 0.0066)	GM - NU	0.29	0.88
E_{rr}	GS	6	0.0038 (0.0031, 0.0046)	GS - GM	0.75	1.00
	GM	9	0.0039 (0.0030, 0.0049)	GS - NU	0.24	0.73
	NU	8	0.0054 (0.0029, 0.0078)	GM - NU	0.29	0.86
$E_{\theta\theta}$	GS	6	0.0029 (0.0017, 0.0041)	GS - GM	0.71	1.00
	GM	9	0.0031 (0.0022, 0.0040)	GS - NU	0.14	0.41
	NU	8	0.0048 (0.0026, 0.0070)	GM - NU	0.16	0.49

Table S8: Comparison of LC strains at 10 mmHg between normal undamaged (NU), glaucoma undamaged and mild-damaged (GM) and glaucoma moderate-damage and severe-damage (GS) groups. * P-value adjusted for multiple comparisons.

Strain Outcome 45 mmHg	Group	Number of Eyes	Estimated Mean Outcome (95% CI)	Pairwise Comparison	p-value	Adjusted* p-value
E_{XX}	GS	6	0.0113 (0.0080, 0.0146)	GS - GM	**0.04**	0.11
	GM	8	0.0075 (0.0064, 0.0085)	GS - NU	0.58	1.00
	NU	8	0.0126 (0.0094, 0.0159)	GM - NU	**0.003**	**0.01**
E_{YY}	GS	6	0.0124 (0.0079, 0.0169)	GS - GM	0.93	1.00
	GM	8	0.0122 (0.0106, 0.0138)	GS - NU	0.54	1.00
	NU	8	0.0146 (0.0093, 0.0199)	GM - NU	0.40	1.00
E_{max}	GS	6	0.0224 (0.0167, 0.0282)	GS - GM	0.40	1.00
	GM	8	0.0200 (0.0183, 0.0217)	GS - NU	0.52	1.00
	NU	8	0.0251 (0.0192, 0.0311)	GM - NU	0.11	0.32
Γ_{max}	GS	6	0.0105 (0.0083, 0.0128)	GS - GM	0.94	1.00
	GM	8	0.0104 (0.0088, 0.0120)	GS - NU	0.62	1.00
	NU	8	0.0116 (0.0080, 0.0152)	GM - NU	0.57	1.00
E_{rr}	GS	6	0.0138 (0.0098, 0.0176)	GS - GM	0.17	0.51
	GM	8	0.0112 (0.0095, 0.0129)	GS - NU	0.78	1.00
	NU	8	0.0147 (0.0096, 0.0197)	GM - NU	0.20	0.61
$E_{\theta\theta}$	GS	6	0.0097 (0.0062, 0.0132)	GS - GM	0.57	1.00
	GM	8	0.0084 (0.0060, 0.0108)	GS - NU	0.32	0.97
	NU	8	0.0122 (0.0086, 0.0158)	GM - NU	0.09	0.26

Table S9: Comparison of LC strains at 45 mmHg between normal undamaged (NU), glaucoma undamaged and mild-damaged (GM) and glaucoma moderate-damage and severe-damage (GS) groups. * P-value adjusted for multiple comparisons.

NU Eyes, 45 mmHg Central Quadrant Strain	Central LC Quadrant (n=8)	Estimated Mean Strain (95% CI)	p-value	Pairwise Comparison	p-value	Adjusted p-value*
E_{XX} (10^{-2})	S	0.0112 (0.0053, 0.0170)	0.16	S-T	0.93	1.00
	T	0.0114 (0.0056, 0.0173)		S-I	0.61	1.00
	I	0.0095 (0.0036, 0.0153)		S-N	0.06	0.34
	N	0.0046 (-0.0013, 0.0104)		T-I	0.55	1.00
				T-N	**0.05**	0.29
				I-N	0.15	0.90
E_{YY} (10^{-2})	S	0.0204 (0.0100, 0.0307)	0.41	S-T	0.27	1.00
	T	0.0147 (0.0043, 0.0250)		S-I	0.14	0.81
	I	0.0126 (0.0022, 0.0229)		S-N	0.16	0.96
	N	0.0130 (0.0027, 0.0234		T-I	0.68	1.00
				T-N	0.75	1.00
				I-N	0.92	1.00
E_{max} (10^{-2})	S	0.0234 (0.0143, 0.0324)	0.27	S-T	0.72	1.00
	T	0.0222 (0.0132, 0.0313)		S-I	0.94	1.00
	I	0.0236 (0.0146, 0.0327)		S-N	0.10	0.59
	N	0.0178 (0.0087, 0.0268)		T-I	0.67	1.00
				T-N	0.19	1.00
				I-N	0.09	0.51
Γ_{max} (10^{-2})	S	0.0074 (0.0031, 0.0118)	**0.04**	S-T	0.36	1.00
	T	0.0090 (0.0046, 0.0133)		S-I	**0.01**	0.03
	I	0.0124 (0.0081, 0.0168)		S-N	0.43	1.00
	N	0.0088 (0.0044, 0.0131)		T-I	**0.05**	0.28
				T-N	0.90	1.00
				I-N	0.04	0.22
E_{rr} (10^{-2})	S	0.0185 (0.0090, 0.0280)	0.17	S-T	0.32	1.00
	T	0.0129 (0.0034, 0.0224)		S-I	0.17	1.00
	I	0.0106 (0.0011, 0.0201)		S-N	**0.03**	0.18
	N	0.0056 (-0.0039, 0.0151)		T-I	0.69	1.00
				T-N	0.21	1.00
				I-N	0.38	1.00
$E_{\theta\theta}$ (10^{-2})	S	0.0135 (0.0071, 0.0199)	0.86	S-T	0.92	1.00
	T	0.0138 (0.0073, 0.0202)		S-I	0.52	1.00
	I	0.0119 (0.0055, 0.0183)		S-N	0.69	1.00
	N	0.0125 (0.0061, 0.0189)		T-I	0.46	1.00
				T-N	0.62	1.00
				I-N	0.81	1.00

Table S10: Comparison of central LC strains averaged within the 4 quadrants at 45 mmHg in the normal undamaged group (NU). Strains were usually smallest in the nasal quadrant, but there was no significant variation after adjusting for multiple comparisons * P-value adjusted for multiple comparisons.

GM Eyes, 45 mmHg Central Quadrant Strain	Central LC Quadrant (n=8)	Estimated Mean Strain (95% CI)	p-value	Pairwise Comparison	p-value	Adjusted p-value*
E_{XX} (10^{-2})	S	0.0036 (-0.0007 0.0079)	0.22	S-T	0.19	1.00
	T	0.0077 (0.0034, 0.0120)		S-I	0.18	1.00
	I	0.0077 (0.0034, 0.0121)		S-N	0.75	1.00
	N	0.0026 (-0.0017, 0.0069)		T-I	0.99	1.00
				T-N	0.11	0.64
				I-N	0.11	0.63
E_{YY} (10^{-2})	S	0.0120 (0.0061, 0.0179)	0.65	S-T	0.91	1.00
	T	0.0118 (0.0058, 0.0177)		S-I	0.84	1.00
	I	0.0116 (0.0056, 0.0175)		S-N	0.27	1.00
	N	0.0095 (0.0036, 0.0154)		T-I	0.93	1.00
				T-N	0.31	1.00
				I-N	0.36	1.00
E_{max} (10^{-2})	S	0.0183 (0.0107, 0.0260)	0.16	S-T	0.48	1.00
	T	0.0202 (0.0126, 0.0279)		S-I	0.62	1.00
	I	0.0197 (0.0120, 0.0273)		S-N	0.17	0.99
	N	0.0146 (0.0070, 0.0223)		T-I	0.84	1.00
				T-N	0.04	0.25
				I-N	0.06	0.39
Γ_{max} (10^{-2})	S	0.0104 (0.0060, 0.0148)	0.21	S-T	0.95	1.00
	T	0.0103 (0.0059, 0.0148)		S-I	0.61	1.00
	I	0.0099 (0.0054, 0.0143)		S-N	0.06	0.38
	N	0.0084 (0.0040, 0.0128)		T-I	0.65	1.00
				T-N	0.07	0.43
				I-N	0.17	1.00
E_{rr} (10^{-2})	S	0.0102 (0.0058, 0.0147)	0.07	S-T	0.67	1.00
	T	0.0091 (0.0047, 0.0136)		S-I	0.53	1.00
	I	0.0118 (0.0074, 0.0162)		S-N	0.05	0.28
	N	0.0050 (0.0005, 0.0094)		T-I	0.30	1.00
				T-N	0.11	0.67
				I-N	**0.01**	0.07
$E_{\theta\theta}$ (10^{-2})	S	0.0058 (0.0004, 0.0113)	0.31	S-T	0.07	0.39
	T	0.0108 (0.0054, 0.0162)		S-I	0.42	1.00
	I	0.0080 (0.0025, 0.0134)		S-N	0.49	1.00
	N	0.0076 (0.0022, 0.0131)		T-I	0.28	1.00
				T-N	0.23	1.00
				I-N	0.91	1.00

Table S11: Comparison of central LC strains averaged within the 4 quadrants at 45 mmHg in the glaucoma no-mild group (GM). Strains were usually smallest in the nasal quadrant, but there was no significant variation after adjusting for multiple comparisons * P-value adjusted for multiple comparisons.

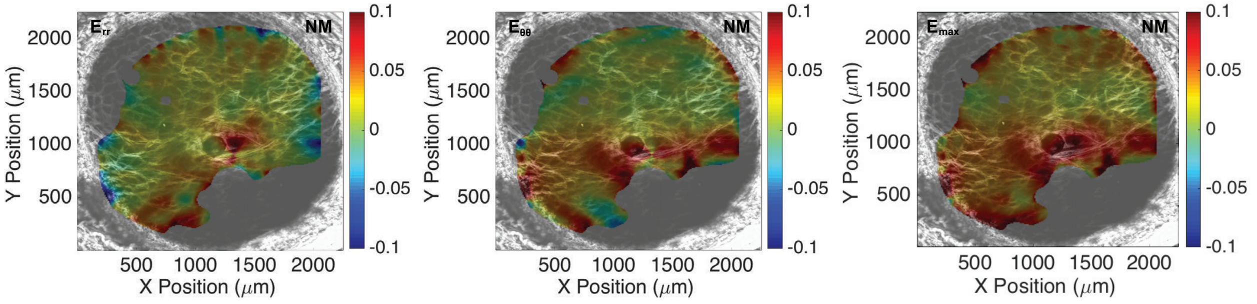

(a) (b) (c)

Figure S2: Strain within the LC of Eye 1 (normal moderately-damaged) for inflation from 5-45 mmHg showing a) radial strain E_{rr}, b) circumferential strain $E_{\theta\theta}$, and c) maximum principal strain E_{max}.

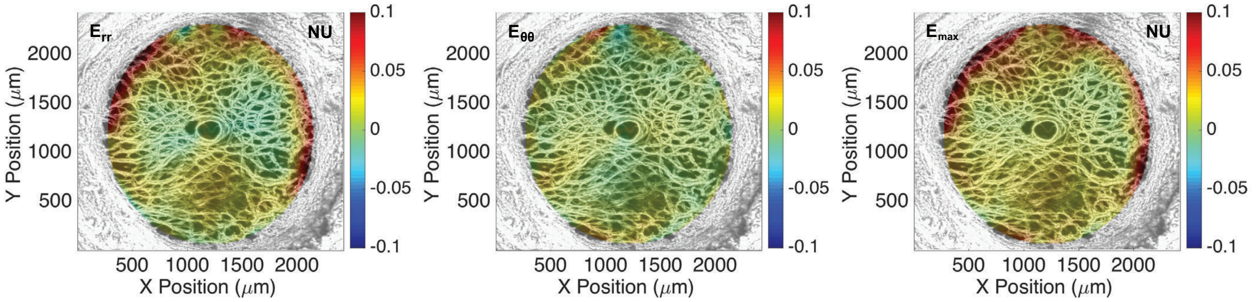

(a) (b) (c)

Figure S3: Strain within the LC of Eye 2 (normal undamaged) for inflation from 5-45 mmHg showing a) radial strain E_{rr}, b) circumferential strain $E_{\theta\theta}$, and c) maximum principal strain E_{max}.

S3 LC Strain Images

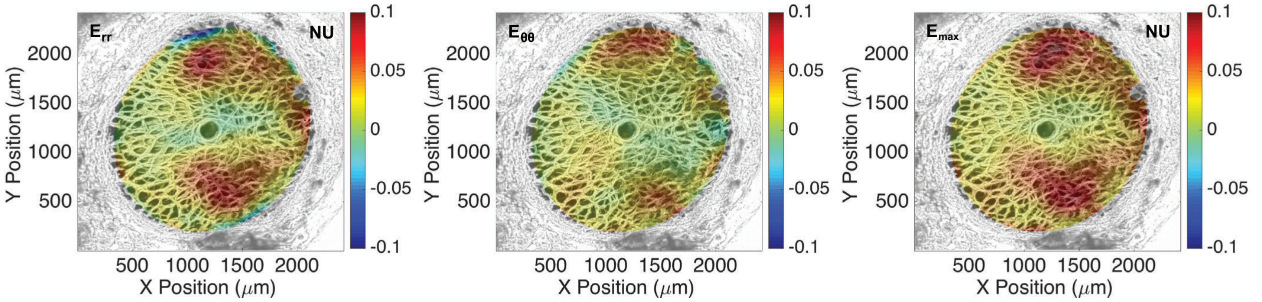

(a) (b) (c)

Figure S4: Strain within the LC of Eye 3 (normal undamaged) for inflation from 5-45 mmHg showing a) radial strain E_{rr}, b) circumferential strain $E_{\theta\theta}$, and c) maximum principal strain E_{max}.

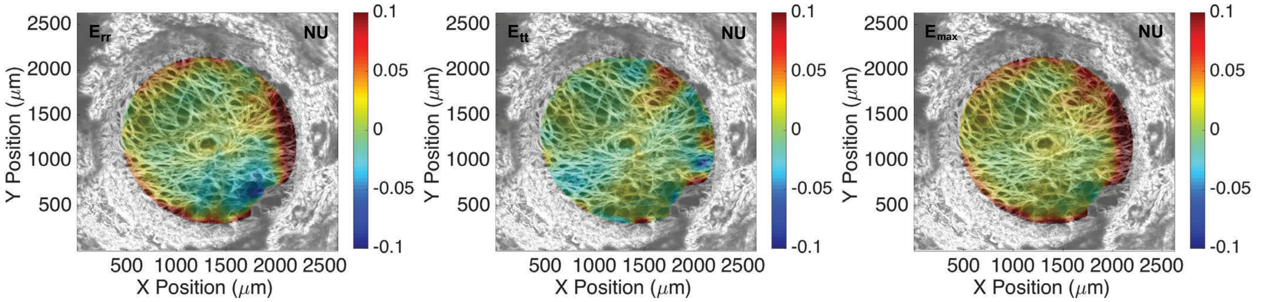

(a) (b) (c)

Figure S5: Strain within the LC of Eye 4 (normal undamaged) for inflation from 5-45 mmHg showing a) radial strain E_{rr}, b) circumferential strain $E_{\theta\theta}$, and c) maximum principal strain E_{max}.

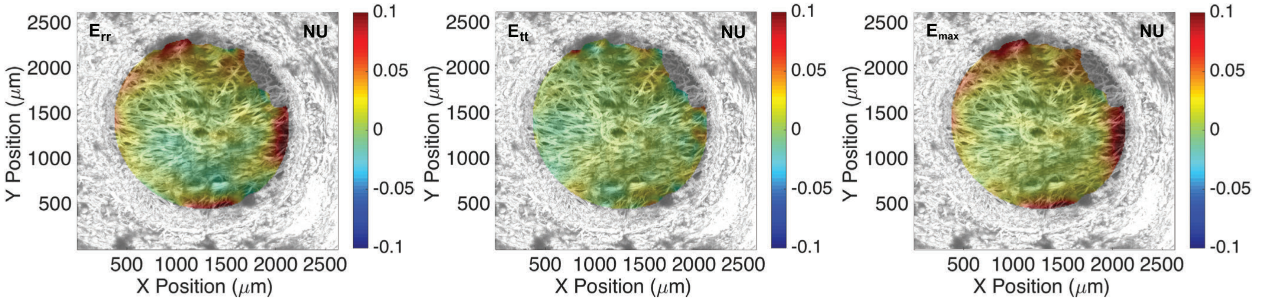

(a) (b) (c)

Figure S6: Strain within the LC of Eye 5 (normal undamaged) for inflation from 5-45 mmHg showing a) radial strain E_{rr}, b) circumferential strain $E_{\theta\theta}$, and c) maximum principal strain E_{max}.

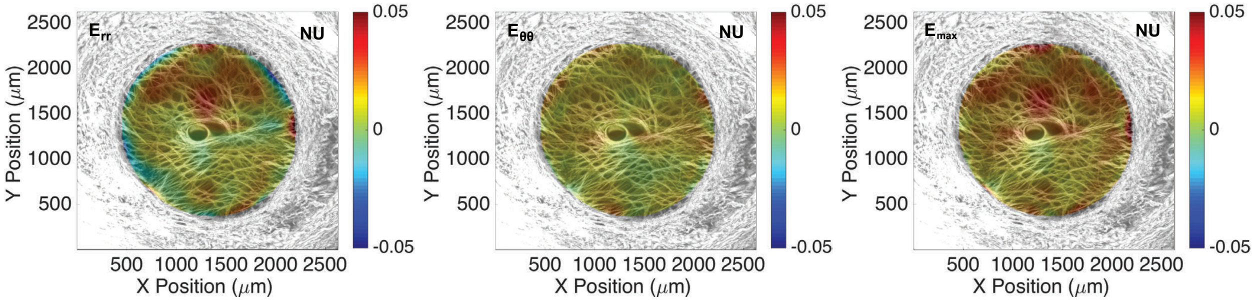

(a) (b) (c)

Figure S7: Strain within the LC of Eye 6 (normal undamaged) for inflation from 5-45 mmHg showing a) radial strain E_{rr}, b) circumferential strain $E_{\theta\theta}$, and c) maximum principal strain E_{max}.

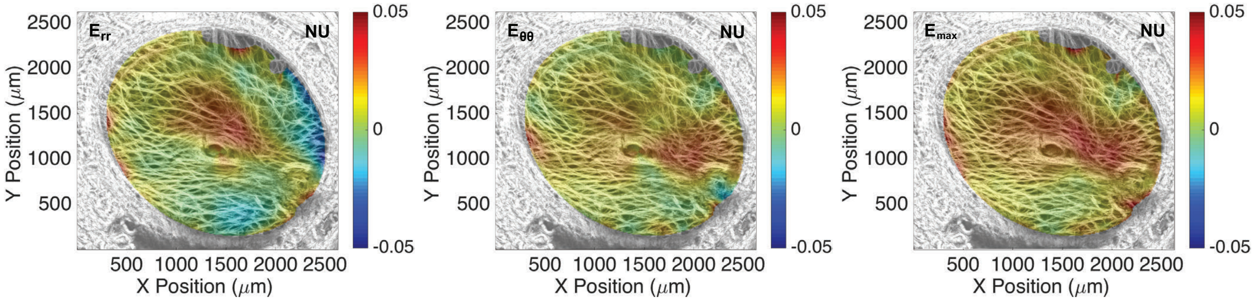

(a) (b) (c)

Figure S8: Strain within the LC of Eye 7 (normal mildly-damaged) for inflation from 5-45 mmHg showing a) radial strain E_{rr}, b) circumferential strain $E_{\theta\theta}$, and c) maximum principal strain E_{max}.

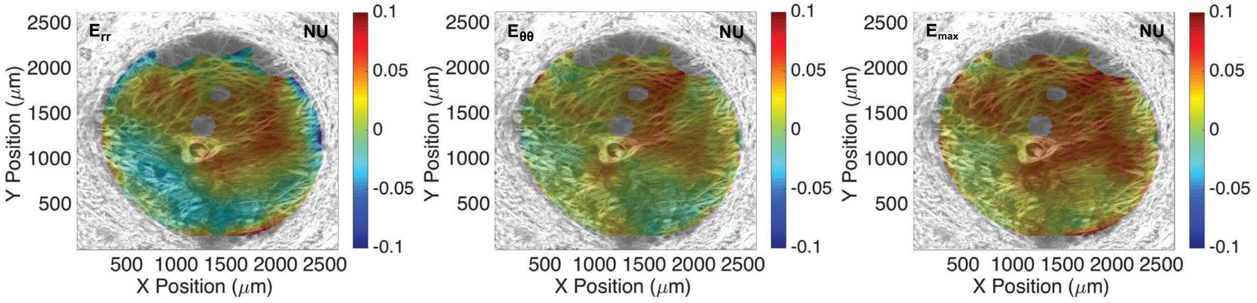

(a) (b) (c)

Figure S9: Strain within the LC of Eye 8 (normal undamaged) for inflation from 5-45 mmHg showing a) radial strain E_{rr}, b) circumferential strain $E_{\theta\theta}$, and c) maximum principal strain E_{max}.

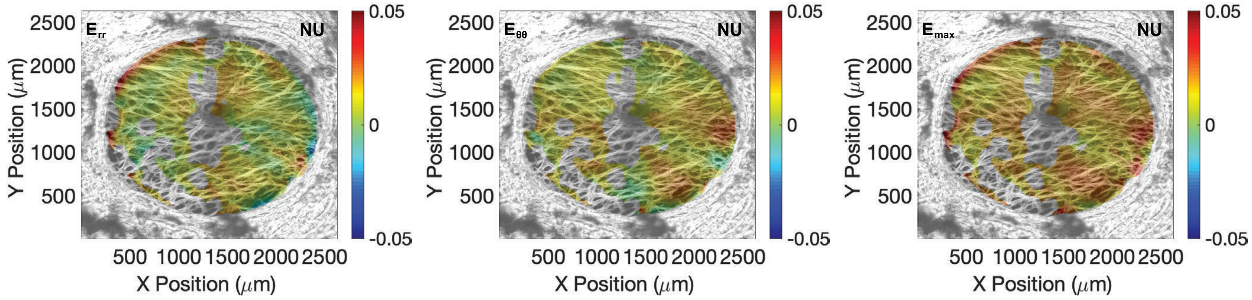

(a) (b) (c)

Figure S10: Strain within the LC of Eye 9 (normal undamaged) for inflation from 5-45 mmHg showing a) radial strain E_{rr}, b) circumferential strain $E_{\theta\theta}$, and c) maximum principal strain E_{max}.

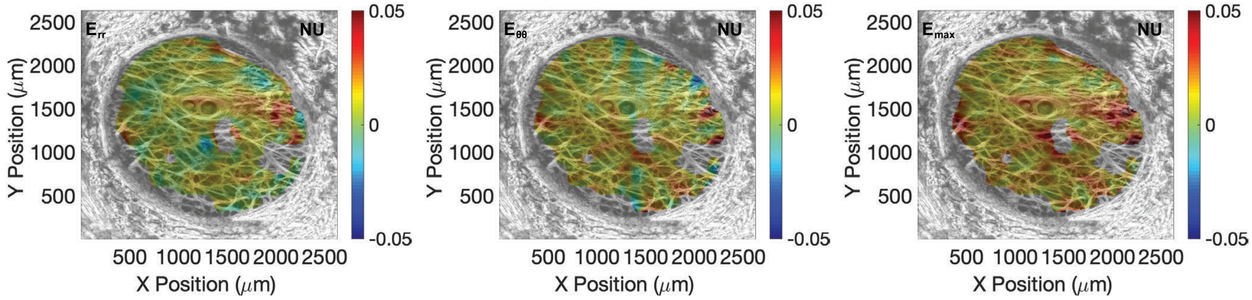

(a) (b) (c)

Figure S11: Strain within the LC of Eye 10 (normal undamaged) for inflation from 5-45 mmHg showing a) radial strain E_{rr}, b) circumferential strain $E_{\theta\theta}$, and c) maximum principal strain E_{max}.

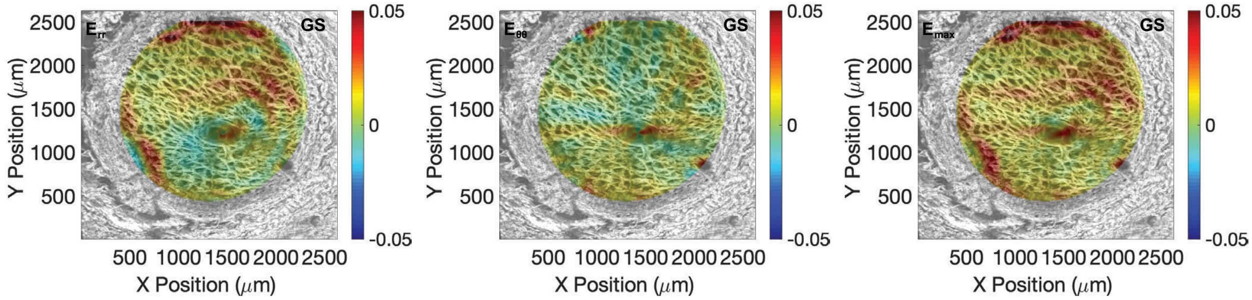

(a) (b) (c)

Figure S12: Strain within the LC of Eye 11 (glaucoma severely-damaged) for inflation from 5-45 mmHg showing a) radial strain E_{rr}, b) circumferential strain $E_{\theta\theta}$, and c) maximum principal strain E_{max}.

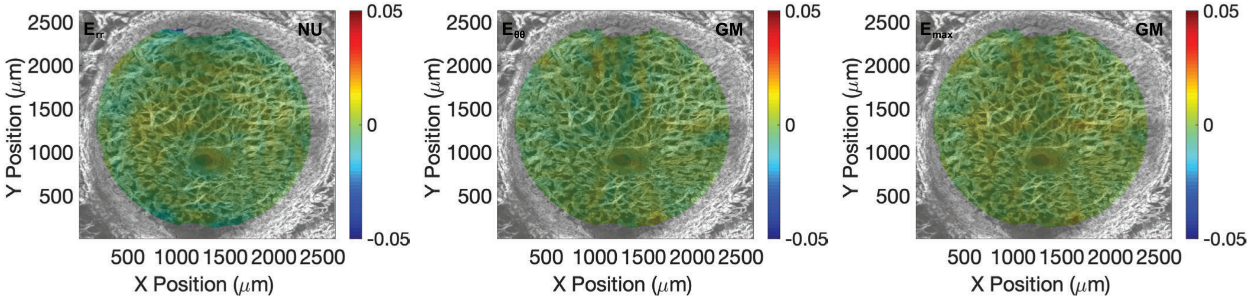

(a) (b) (c)

Figure S13: Strain within the LC of Eye 12 (glaucoma mildly-damaged) for inflation from 5-10 mmHg (45 mmHg images had stitching errors) showing a) radial strain E_{rr}, b) circumferential strain $E_{\theta\theta}$, and c) maximum principal strain E_{max}.

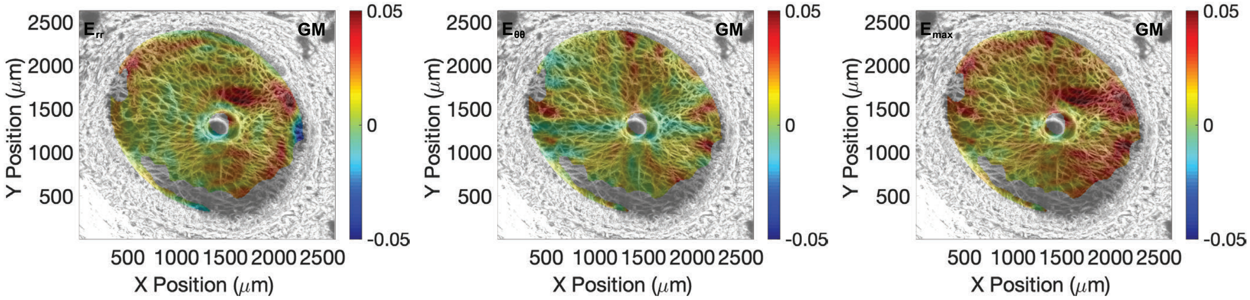

(a) (b) (c)

Figure S14: Strain within the LC of Eye 13 (glaucoma undamaged) for inflation from 5-45 mmHg showing a) radial strain E_{rr}, b) circumferential strain $E_{\theta\theta}$, and c) maximum principal strain E_{max}.

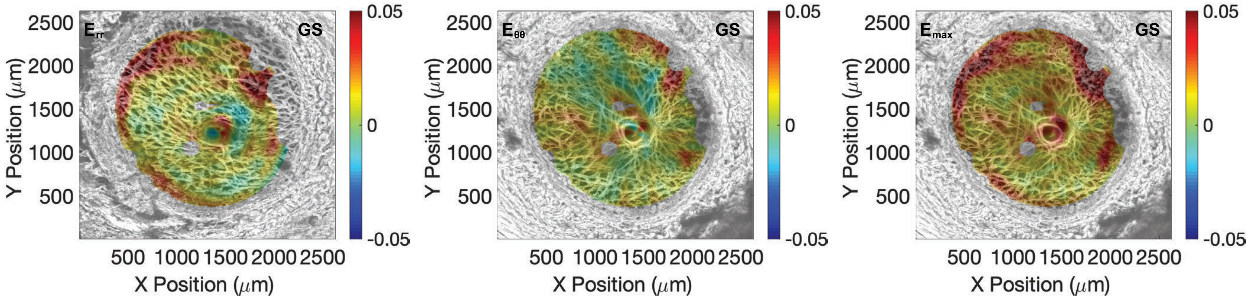

(a) (b) (c)

Figure S15: Strain within the LC of Eye 14 (glaucoma severely-damaged) for inflation from 5-45 mmHg showing a) radial strain E_{rr}, b) circumferential strain $E_{\theta\theta}$, and c) maximum principal strain E_{max}.

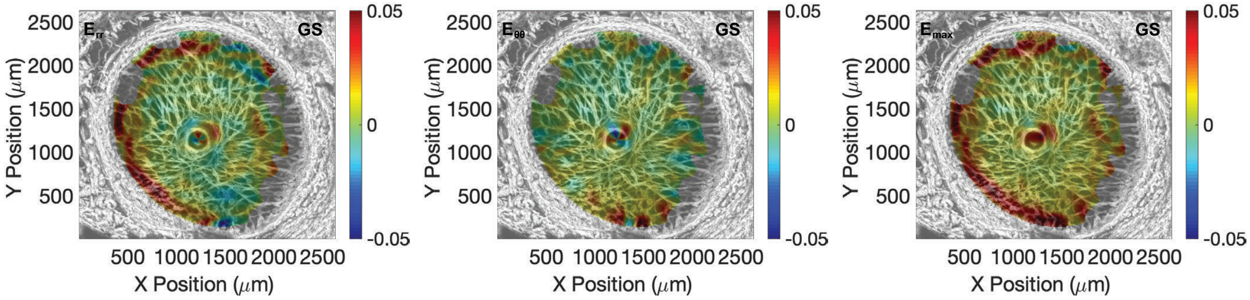

(a) (b) (c)

Figure S16: Strain within the LC of Eye 15 (glaucoma moderately-damaged) for inflation from 5-45 mmHg showing a) radial strain E_{rr}, b) circumferential strain $E_{\theta\theta}$, and c) maximum principal strain E_{max}.

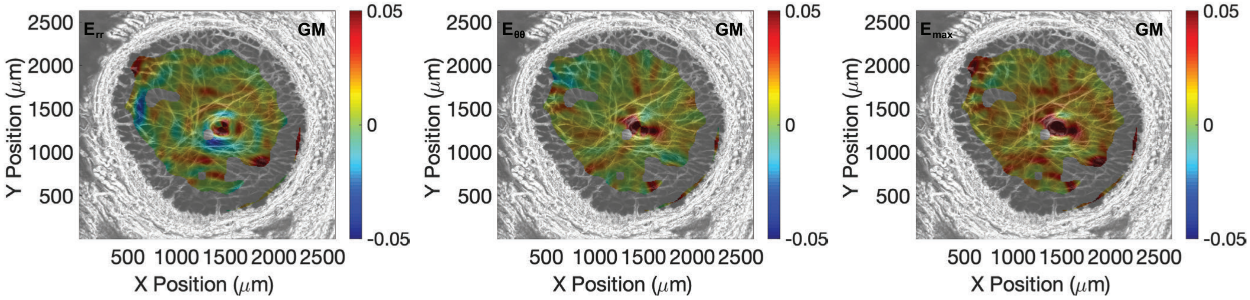

(a) (b) (c)

Figure S17: Strain within the LC of Eye 16 (glaucoma undamaged) for inflation from 5-45 mmHg showing a) radial strain E_{rr}, b) circumferential strain $E_{\theta\theta}$, and c) maximum principal strain E_{max}.

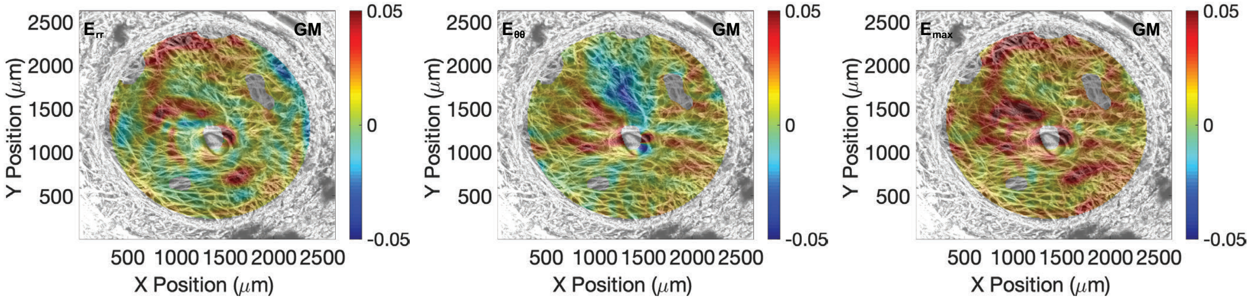

(a) (b) (c)

Figure S18: Strain within the LC of Eye 17 (glaucoma undamaged) for inflation from 5-45 mmHg showing a) radial strain E_{rr}, b) circumferential strain $E_{\theta\theta}$, and c) maximum principal strain E_{max}.

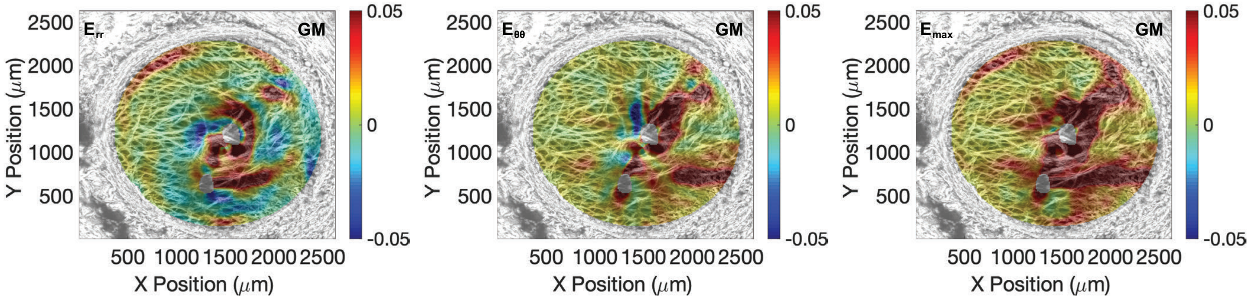

(a) (b) (c)

Figure S19: Strain within the LC of Eye 18 (glaucoma undamaged) for inflation from 5-45 mmHg showing a) radial strain E_{rr}, b) circumferential strain $E_{\theta\theta}$, and c) maximum principal strain E_{max}.

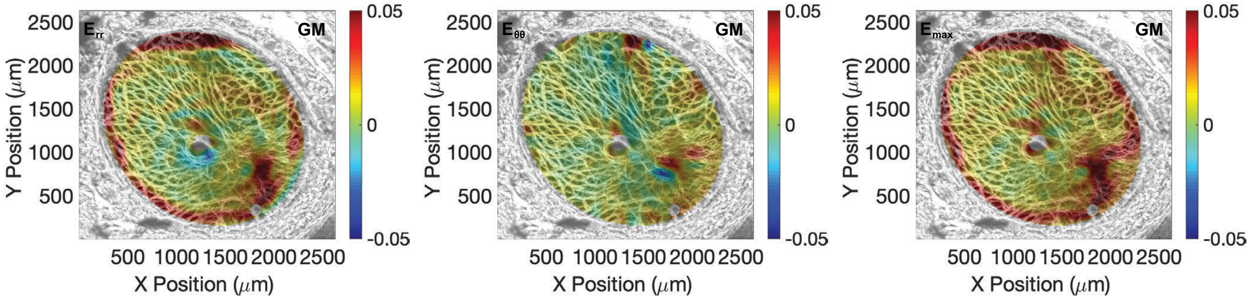

(a) (b) (c)

Figure S20: Strain within the LC of Eye 19 (glaucoma undamaged) for inflation from 5-45 mmHg showing a) radial strain E_{rr}, b) circumferential strain $E_{\theta\theta}$, and c) maximum principal strain E_{max}.

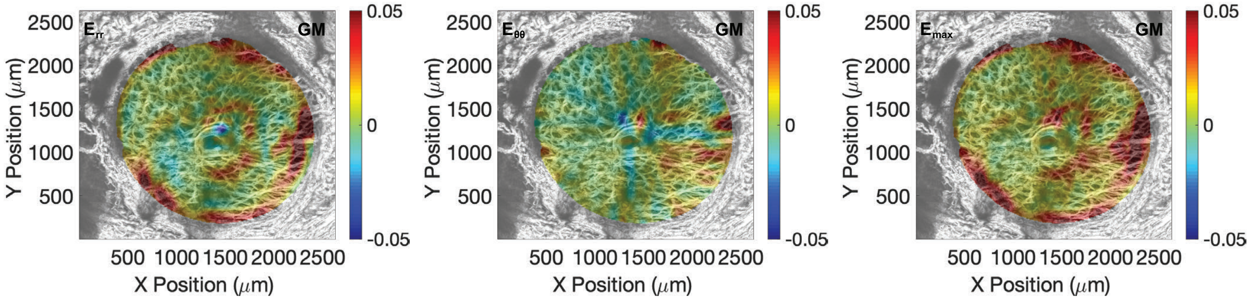

(a) (b) (c)

Figure S21: Strain within the LC of Eye 20 (glaucoma undamaged) for inflation from 5-45 mmHg showing a) radial strain E_{rr}, b) circumferential strain $E_{\theta\theta}$, and c) maximum principal strain E_{max}.

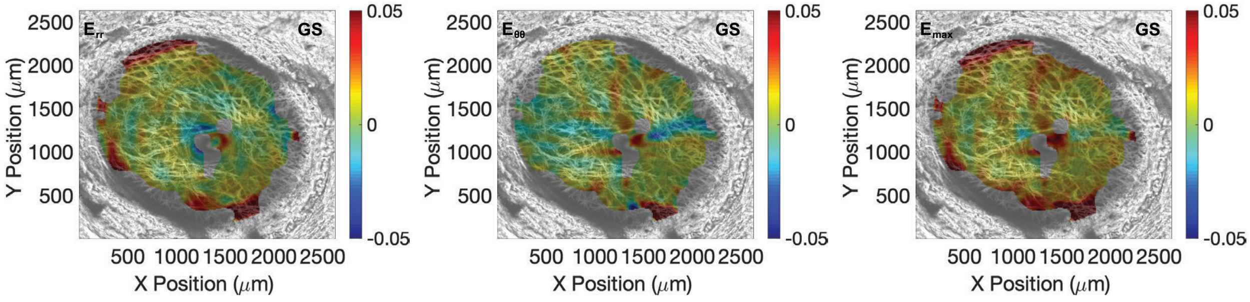

(a) (b) (c)

Figure S22: Strain within the LC of Eye 21 (glaucoma moderately-damaged) for inflation from 5-45 mmHg showing a) radial strain E_{rr}, b) circumferential strain $E_{\theta\theta}$, and c) maximum principal strain E_{max}.

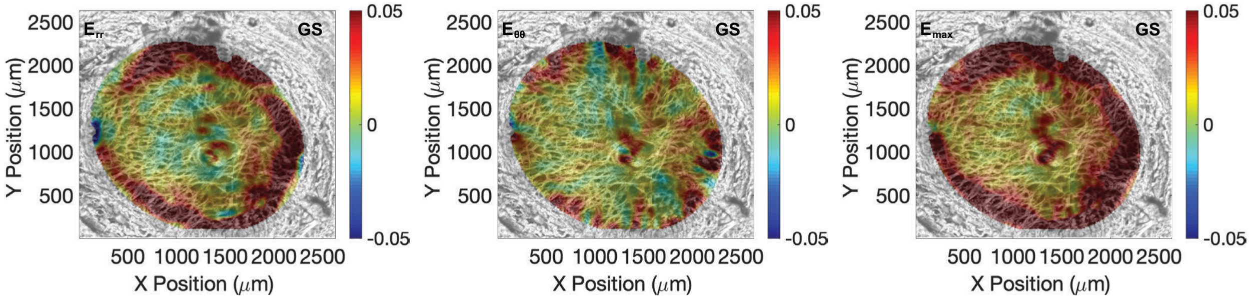

(a) (b) (c)

Figure S23: Strain within the LC of Eye 22 (glaucoma severely-damaged) for inflation from 5-45 mmHg showing a) radial strain E_{rr}, b) circumferential strain $E_{\theta\theta}$, and c) maximum principal strain E_{max}.

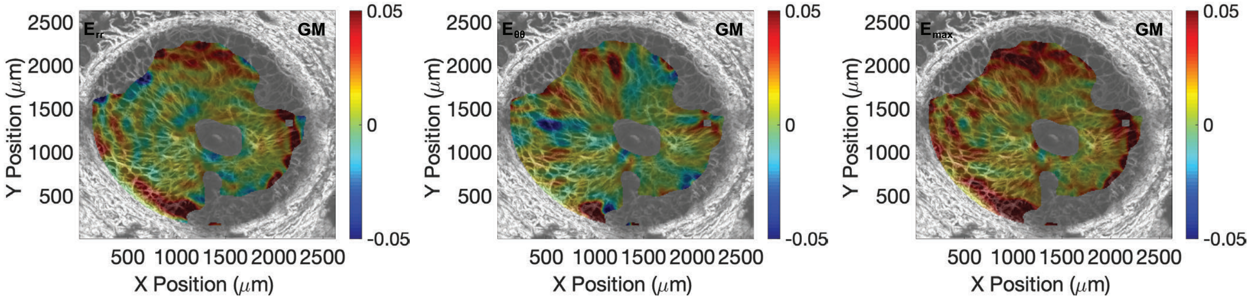

(a) (b) (c)

Figure S24: Strain within the LC of Eye 23 (glaucoma mildly-damaged) for inflation from 5-45 mmHg showing a) radial strain E_{rr}, b) circumferential strain $E_{\theta\theta}$, and c) maximum principal strain E_{max}.

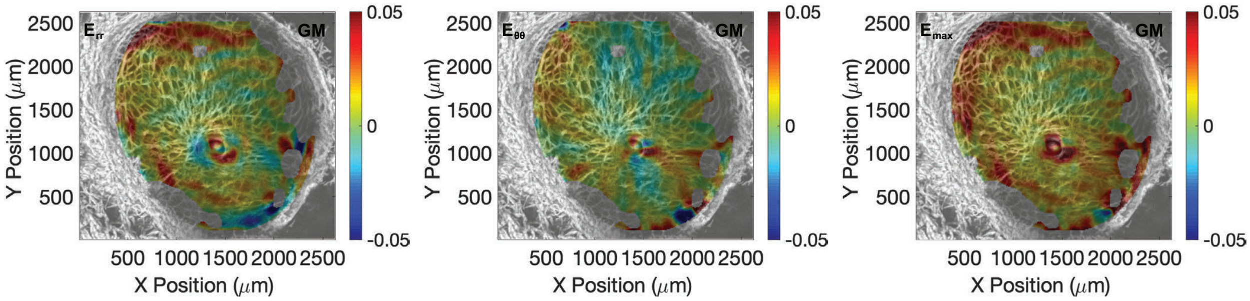

(a) (b) (c)

Figure S25: Strain within the LC of Eye 24 (glaucoma mildly-damaged) for inflation from 5-45 mmHg showing a) radial strain E_{rr}, b) circumferential strain $E_{\theta\theta}$, and c) maximum principal strain E_{max}.

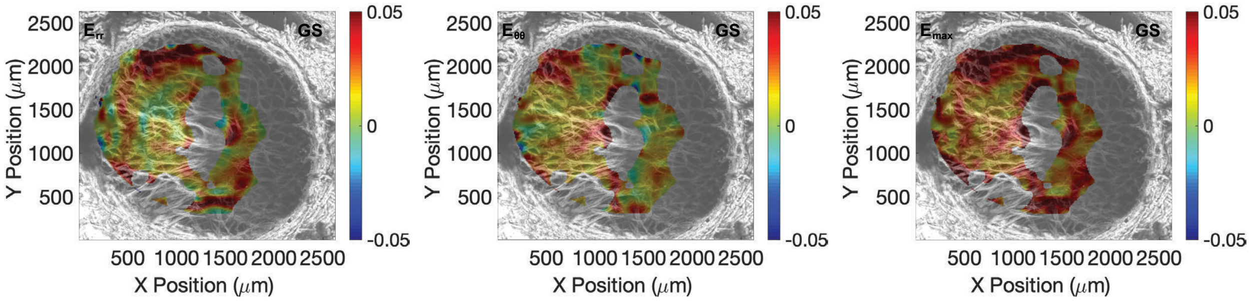

(a) (b) (c)

Figure S26: Strain within the LC of Eye 25 (glaucoma moderately-damaged) for inflation from 5-45 mmHg showing a) radial strain E_{rr}, b) circumferential strain $E_{\theta\theta}$, and c) maximum principal strain E_{max}.

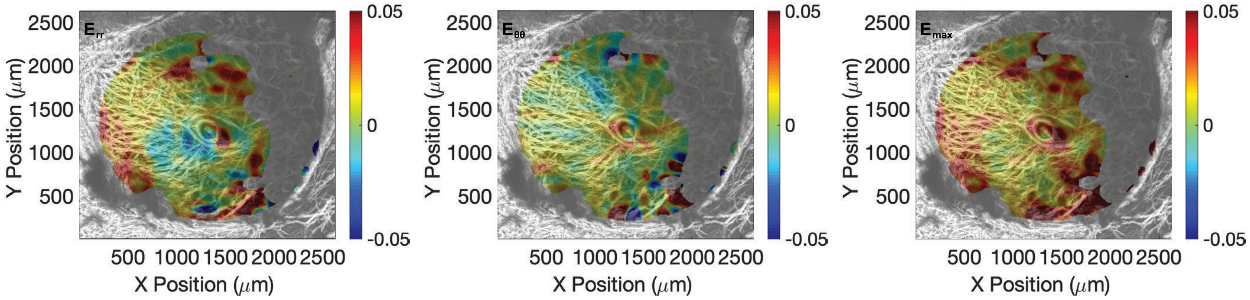

(a) (b) (c)

Figure S27: Strain within the LC of Eye 26 (glaucoma unknown damage) for inflation from 5-45 mmHg showing a) radial strain E_{rr}, b) circumferential strain $E_{\theta\theta}$, and c) maximum principal strain E_{max}.
